# DDX24 Mutation Alters NPM1 Phase Behavior and Disrupts Nucleolar Homeostasis in Vascular Malformations

**DOI:** 10.7150/ijbs.84097

**Published:** 2023-08-06

**Authors:** Haopei Zhang, Qiuyue Chen, Qianqian Zhang, Hairun Gan, Hanjie Li, Shoudeng Chen, Hong Shan, Pengfei Pang, Huanhuan He

**Affiliations:** 1 Guangdong Provincial Engineering Research Center of Molecular Imaging, The Fifth Affiliated Hospital of Sun Yat-sen University, Zhuhai, China 519000.; 2 Center for Interventional Medicine, The Fifth Affiliated Hospital of Sun Yat-sen University, Zhuhai, China 519000.; 3 Department of Interventional Medicine, The Fifth Affiliated Hospital of Sun Yat-sen University, Zhuhai, China 519000.; 4 Department of Radiology, The Fifth Affiliated Hospital of Sun Yat-sen University, Zhuhai, Guangdong Province, China 519000.

**Keywords:** DEAD-box helicase 24, liquid-liquid phase separation, nucleolar homeostasis, NPM1, vascular malformation

## Abstract

Point mutations in the DEAD-box helicase DDX24 are associated with vascular malformations such as multi-organ venous and lymphatic defect (MOVLD) syndrome and Budd-Chiari syndrome, with the pathogenesis largely uncharacterized. DDX24 is mainly located in the nucleolus, where nucleophosmin (NPM1) regulates nucleolar homeostasis via liquid-liquid phase separation (LLPS). However, the connection between DDX24 and NPM1 in vascular malformation remains elusive. Here we demonstrated that DDX24 formed biomolecular condensates in vitro and the mutated DDX24 protein, DDX24^E271K^, partitioned less into the nucleoli in tissues from patients with MOVLD syndrome and cultured endothelial cells (ECs), altering nucleolar morphology. Furthermore, DDX24 was directly associated with NPM1 to regulate its phase behavior as a client in the nucleolar granular component (GC). Functionally, we showed that DDX24 was essential in maintaining nucleolar homeostasis of ECs and that either mutation or knockdown of DDX24 led to the dysfunction of ribosome biogenesis and the elevated capability of cell migration and tube formation. Our findings illustrate how DDX24 mutation affects nucleolar structure and function by regulating the phase behavior of NPM1 in the setting of vascular malformation.

## Introduction

Nucleoli are distinct membrane-less nuclear bodies that are primarily dedicated to ribosome biogenesis and play an essential role in ribonucleoprotein (RNP) complex assembly, DNA damage response, cell-cycle control, and stress response^1^-^3^. Hundreds of molecules reside in the nucleolus maintained by an organized multilayer structure composed of the fibrillar center (FC), the dense fibrillar component (DFC), and the granular component (GC)^4^,^5^. While supporting the architecture, these molecules display constant fluidity and exchange with the surrounding nucleoplasm to sustain nucleolar homeostasis^6^-^8^.

Progress into the biophysical nature of nucleolus has revealed that it forms and maintains through liquid-liquid phase separation (LLPS)^9^-^12^. This model tightly couples nucleolar material properties to its diverse functions. For instance, when cells are under prolonged heat stress, reduced protein mobility in the nucleolus deteriorates the capacity to restore misfolded proteins^13^. Such dysfunction of the nucleolus, or loss of nucleolar homeostasis, prompted by altered nucleolar material state may contribute to disease pathology. In the setting of neurodegenerative disease, aberrantly expressed dipeptide-repeat polypeptides disrupt NPM1 phase separation and its proper function in ribosome assembly to induce cellular toxicity^14^. These mechanistic findings prompt us to revisit the pathology of many diseases associated with malfunctions of nucleolar proteins in the view of nucleolar phase behavior.

Vascular malformations are a heterogeneous group of disorders characterized by altered endothelial cell (EC) proliferation, migration, and viability that are often caused by genetic mutations^15^,^16^. However, the link between nucleolar homeostasis and endothelial function in the settings of vascular malformations remains missing. Previously, we identified a particular type of vascular malformations termed multi-organ venous and lymphatic defect syndrome (MOVLD)^17^, with which patients exhibited life-threatening symptoms and had no effective treatment options. Our work associated the p.Glu271Lys mutation of *DDX24* (DDX24^E271K^) with this disease and demonstrated that DDX24 knockdown promoted endothelial cell migration.

DDX24 belongs to a large ATP-dependent RNA helicase family that many of its members contain intrinsically disordered regions (IDRs) and can undergo LLPS to regulate RNA-containing phase-separated organelles^18^. It is a nucleolar protein ubiquitously expressed across human tissues^19^,^20^. DDX24 has been identified as a common essential gene from viability screens on various cell lines^21^, and its deficiency causes embryonic lethality in mouse^22^. Vital as it may be, it has not been reported whether DDX24 can undergo phase separation or participate in the maintenance of nucleolar homeostasis. Moreover, the mechanism underlying *DDX24* mutation-mediated vascular malformation is still inexplicit.

Here, we investigated the alterations of nucleolar morphology and LLPS properties, and the functional consequences caused by DDX24^E271K^ in vitro and in cells. We demonstrated that DDX24 was essential in maintaining nucleolar homeostasis by controlling the phase behavior of its associated nucleolar protein NPM1. Our findings identify a novel mechanism of vascular malformations, which provides promising diagnostic markers and therapeutic targets for such diseases.

## Results

### DDX24 mutation or deficiency alters nucleolar morphology

**Figure 1:**
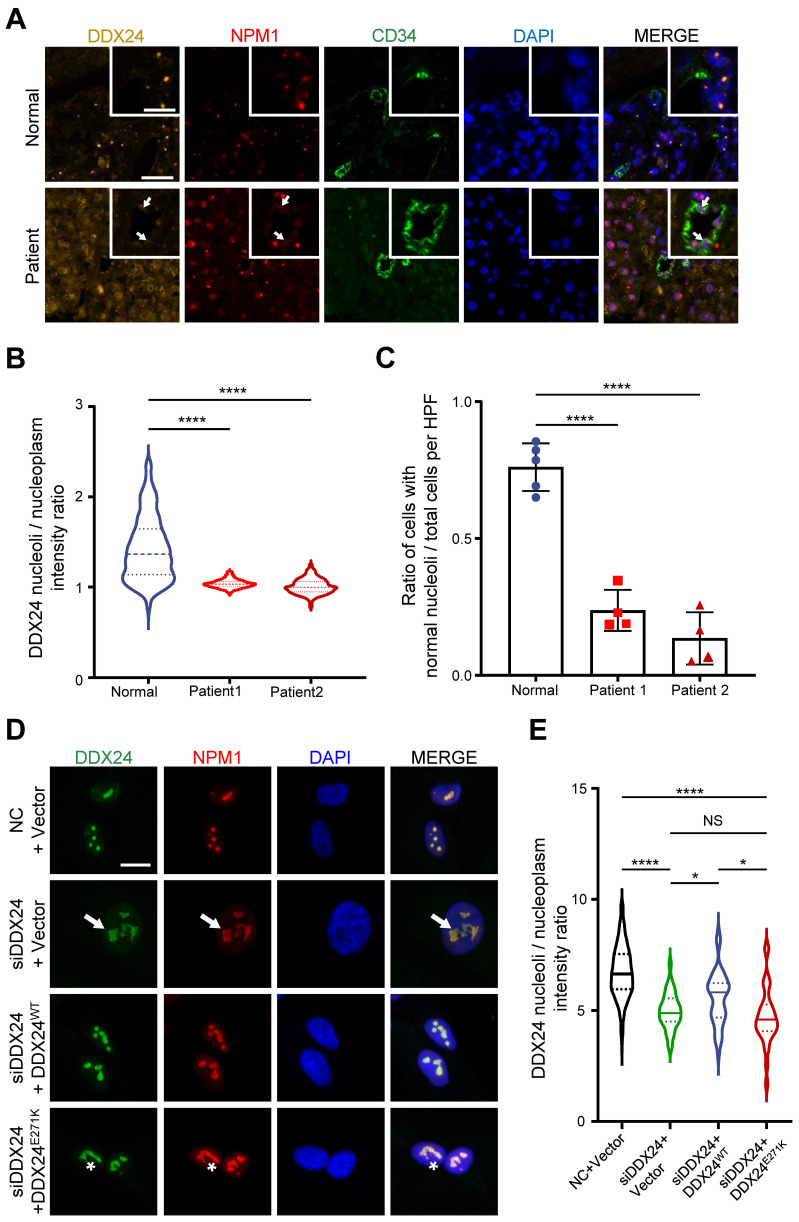
** Abnormal nucleolar phenotype observed in patient samples and cells with *DDX24* p.Glu271Lys mutation. (A)** Representative immunofluorescence images of DDX24 (yellow), NPM1 (red) and CD34 (green) in liver FFPE sections obtained from MOVLD patient. Scale bar 20 μm. Inserted graph represents a higher power field, Scale bar 10 μm. DAPI (blue) stains DNA. Arrow indicates unstructured nucleoli. **(B)** Quantification of the ratio of DDX24 nucleoli intensity / DDX24 nucleoplasm intensity in individual cell from liver FFPE sections. **(C)** Quantification of the ratio of cells with normal nucleoli/total cells per high-power field (HPF) from liver FFPE sections. **(D, E)** Representative immunofluorescence images of DDX24 (green), NPM1 (red) in HUVECs (D) and quantification of the ratio of DDX24 nucleoli intensity / DDX24 nucleoplasm intensity in individual cell (E) from the knock-down and re-expression assay; nucleoli / nucleoplasm intensity ratio was quantified as DDX24 mean fluorescence intensity of nucleoli versus nucleoplasm. Scale bar 20 μm. Arrow indicates enlarged nucleoli in DDX24 knock-down cells, asterisk indicates reduced DDX24 nucleoli / nucleoplasm intensity ratio. Data in (B, E) show the median, quartiles, and maximum / minimum values; data in (C) are shown as mean values ± s.d. *n* > 500 cells per sample (B); *n* > 4 HPFs per sample (C); *n* > 35 cells for each group pooled from 2 independent experiments (E). Kruskal-Wallis test with Dunn's multiple comparisons test was used, except for (C), which used ordinary one-way ANOVA with Dunnett's multiple comparison test. NS, not significant; *P≤ 0.05, **** *P* ≤ 0.0001.

Previously we identified a *DDX2*4 mutation (DDX24^E271K^) to be linked to a particular type of vascular malformations, MOVLD^17^. Upon immunostaining of DDX24 on the liver biopsy of MOVLD patients, we observed that the nucleolar distribution of DDX24 protein was dispersed (**Figure 1A-B**). This was accompanied by the appearance of unstructured nucleoli with undefined boundaries as shown by nebulous NPM1 staining (**Figure 1C**), implying loss of nucleolar homeostasis.

To validate the relationship between DDX24 and nucleolar morphology in vitro, we generated a cellular model by knocking down DDX24 in human umbilical vein endothelial cells (HUVECs) and ectopically expressing DDX24^WT^ or DDX24^E271K^ (**Supplemental Figure 1A**). Upon DDX24 knockdown, the size of the nucleolus enlarged, and the nucleolar deformity was prominent (**Figure 1D**). Notably, the intensity ratio of DDX24 in nucleoli/nucleoplasm dropped significantly upon DDX24 deficiency, which could be rescued by reintroducing DDX24^WT^ but not by DDX24^E271K^ (**Figure 1E**), in line with the findings from patient tissues. Simply overexpressing DDX24^WT^ also affected the nucleoli/nucleoplasm intensity ratio of DDX24 (**Supplemental Figure 1B**). The average size and number of the nucleoli slightly fluctuated with the expression of DDX24 (**Supplemental Figure 1C-D**). Together, these results highlight the contribution of DDX24 to the maintenance of normal nucleolar morphology.

### DDX24 undergoes liquid-liquid phase separation

**Figure 2:**
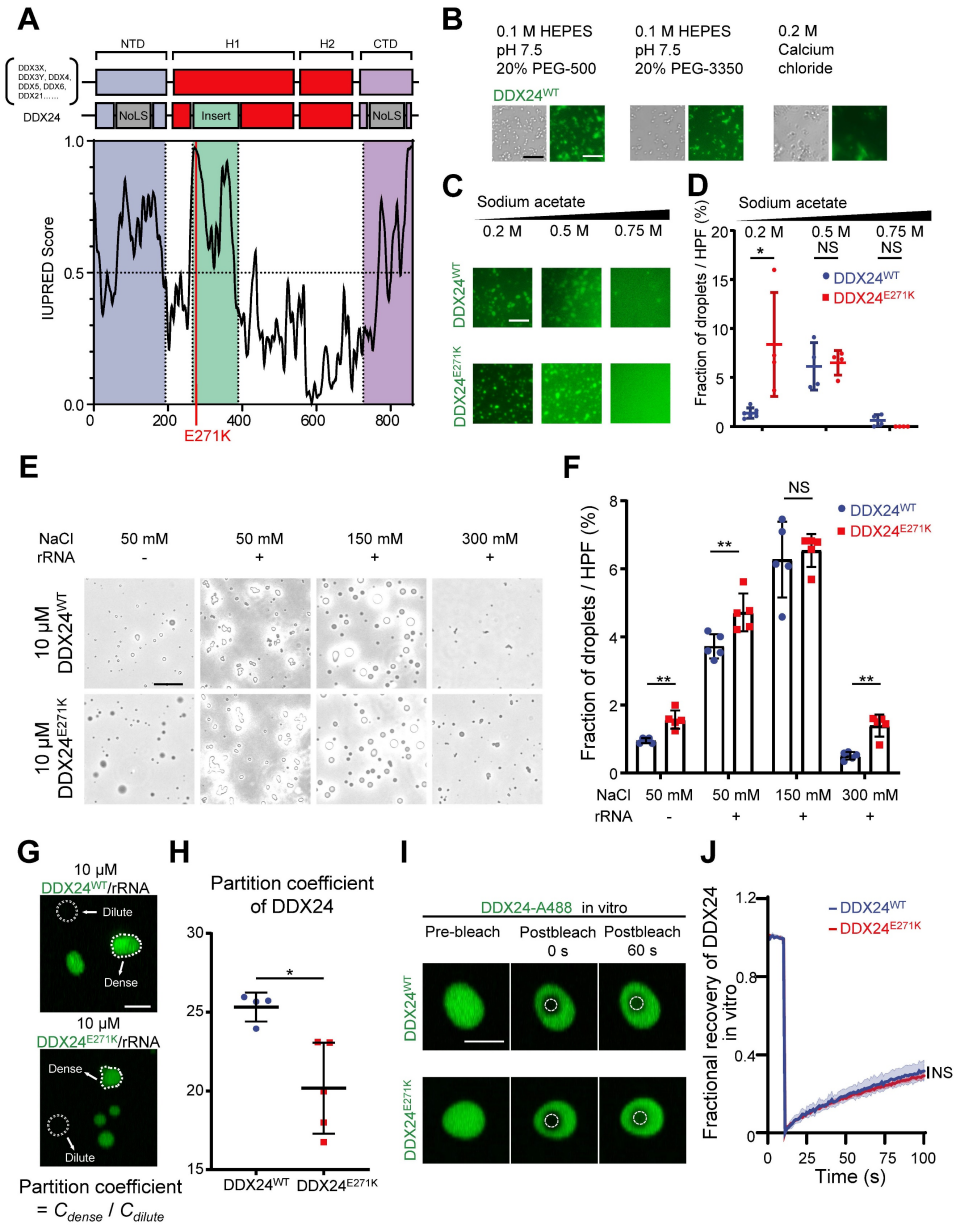
** DDX24 undergoes liquid-liquid phase separation and forms gel-like condensates in vitro. (A)** Domain organization and Analysis of intrinsically disordered domain of DDX24, red line indicates the position of p.Glu271Lys mutation (E271K). **(B)** Representative images of 4 μM DDX24^WT^ protein (Alexa Fluor 488) condensate formation in the buffer condition as noted. Scale bar 50 μm. **(C)** Representative images of 4 μM DDX24^WT^ protein (Alexa Fluor 488) or 4 μM DDX24^E271K^ protein (Alexa Fluor 488) condensate formation in buffers containing the indicated sodium acetate concentration. Scale bar 50 μm. **(D)** Quantification of the extent of droplet formation of DDX24^WT^ or DDX24^E271K^ from images in (C). **(E)** Representative images of 10 μM DDX24^WT^ protein or 10 μM DDX24^E271K^ protein droplet formation in a Tris-NaCl LLPS buffer (20 mM Tris, 1 mM TCEP, pH 8.0) containing the indicated NaCl concentration, with or without the presence of rRNA (100 μg/mL). Scale bar 20 μm. **(F)** Quantification of the extent of droplet formation of DDX24^WT^ or DDX24^E271K^ from images in (E). **(G, H)** Representative confocal images of 10 μM DDX24^WT^ protein (Alexa Fluor 488) or 10 μM DDX24^E271K^ protein (Alexa Fluor 488) droplet formation with rRNA (100 μg/mL) in a Tris-NaCl LLPS buffer (20 mM Tris, 150 mM NaCl, 1 mM TCEP, pH 8.0) (G) and the quantification of partition coefficient for the two DDX24 constructs (H), partition coefficient were quantified as client mean fluorescence intensity of dense phase versus dilute phase as outlined by dotted line in (G). Scale bar 10 μm. **(I)** Confocal microscopy images of DDX24^WT^-A488 or DDX24^E271K^-A488 droplets before and after photobleached in LLPS buffer same as (G). Scale bar 5 μm. **(J)** FRAP recovery curves of DDX24^WT^-A488 or DDX24^E271K^-A488 in droplets of (I); ROI = 1 µm circular area in the center of the droplet outlined by dotted line. Data are shown as mean values ± s.d. Images in (B, C, E, G, I) are representative of 2 independent experiments; *n* ≥ 4 HPFs for each group pooled from 2 independent experiments (D, F, H); *n* ≥ 5 droplets for each group (J). Mann-Whitney test with Bonferroni-Dunn's multiple comparisons test for (D, F); Two-tailed Student's t-test for (H); Two-tailed Student's t-test was used to test the differences in t-half from FRAP curve fitting for (J). NS, not significant; *P≤ 0.05, **P≤ 0.01, **** *P* ≤ 0.0001.

According to previous reports on other DEAD-box proteins that can undergo LLPS^18^,^23^-^25^, we suspected that DDX24 might possess this property. First, we visualized the structure of DDX24 using AlphaFold2 and discovered a low-confidence region spanning from 250-380 that was associated with intrinsical disorder (**Supplemental Figure 2A-B**). Subsequently, we performed sequence analysis on DDX24 protein against other DEAD-box proteins by a protein disorder predictor, IUPRED^26^, and found that the N-terminal domain (NTD), the C-terminal domain (CTD), and the low-confidence region all harbor potential IDRs (**Figure 2A, Supplemental Figure 2C**). Importantly, E271K mutation was located in the IDR of DDX24, suggesting a possible perturbation of its phase separation properties.

To assess the ability of DDX24 to undergo phase separation in vitro, we performed an LLPS screening test with purified full-length DDX24^WT^ and DDX24^E271K^ proteins labeled with Alexa Fluor 488.

Interestingly, DDX24 was prone to condense into a fiber or gel-like structure in low ionic strength or crowding agent conditions (**Figure 2B**). However, at higher ionic strength, the condensates of both DDX24^WT^ and DDX24^E271K^ were disrupted in an ionic strength-dependent manner** (Figure 2C)**, suggesting that homotypic condensation of DDX24 was primarily driven by electrostatic interactions. In addition, we observed that the total area of the condensates formed by DDX24^E271K^ was slightly higher than wildtype at a low ionic strength **(Figure 2D)**. Other buffer conditions were screened and both wildtype and mutant DDX24 behaved alike (**Supplemental Figure 3A**). Thus, DDX24 could form homotypic gel-like condensates mediated by intermolecular electrostatic interactions in vitro, but wildtype and mutant DDX24 performed similarly.

Since the interactions between RNA binding proteins (RBPs) and RNA molecules have been implicated in facilitating LLPS^27^-^29^, we tested whether rRNA would alter the phase separation of the RNA helicase DDX24. Indeed, 100 μg/mL rRNA improved the phase separation of DDX24 condensates significantly (**Figure 2E**). Even though the total area of the heterotypic condensates formed by DDX24^E271K^/rRNA was slightly higher than that by the wildtype (**Figure 2F**), no major difference was observed in the turbidity between DDX24^WT^/rRNA and DDX24^E271K^/rRNA condensates (**Supplemental Figure 3B-C**). However, the partition coefficient, calculated by the ratio of fluorescence intensity in the dense versus dilute phases, was significantly lower for the DDX24^E271K^ (**Figure 2G-H**), echoing the decreased nucleoli/nucleoplasm ratio in cells.

Next, we examined the dynamic properties of the DDX24/rRNA condensates. In vitro fluorescence recovery after photobleaching (FRAP) analysis showed that both DDX24^WT^ and DDX2^E271K^ were relatively less dynamic inside the condensates, with 25% fluorescence recovery within 90 seconds, indicating a gel-like state (**Figure 2I-J).** To examine the LLPS properties of DDX24 in cells, we performed live-cell FRAP in HUVECs overexpressing EGFP-labeled DDX24 (either DDX24^WT^ or DDX24^E271K^)**.** Although the recovery speed of both DDX24 variants in cells was much faster than that in vitro, DDX24^E271K^ and DDX24^WT^ yielded similar results (**Supplemental Figure 3D-E**), suggesting that the mutation did not influence the internal dynamics of DDX24. Collectively, our results demonstrate that DDX24 can form condensates with or without rRNA through LLPS and DDX24^E271K^ exhibits altered partition into the condensates.

### DDX24 interacts with NPM1 in the granular component of nucleolus as a client

**Figure 3:**
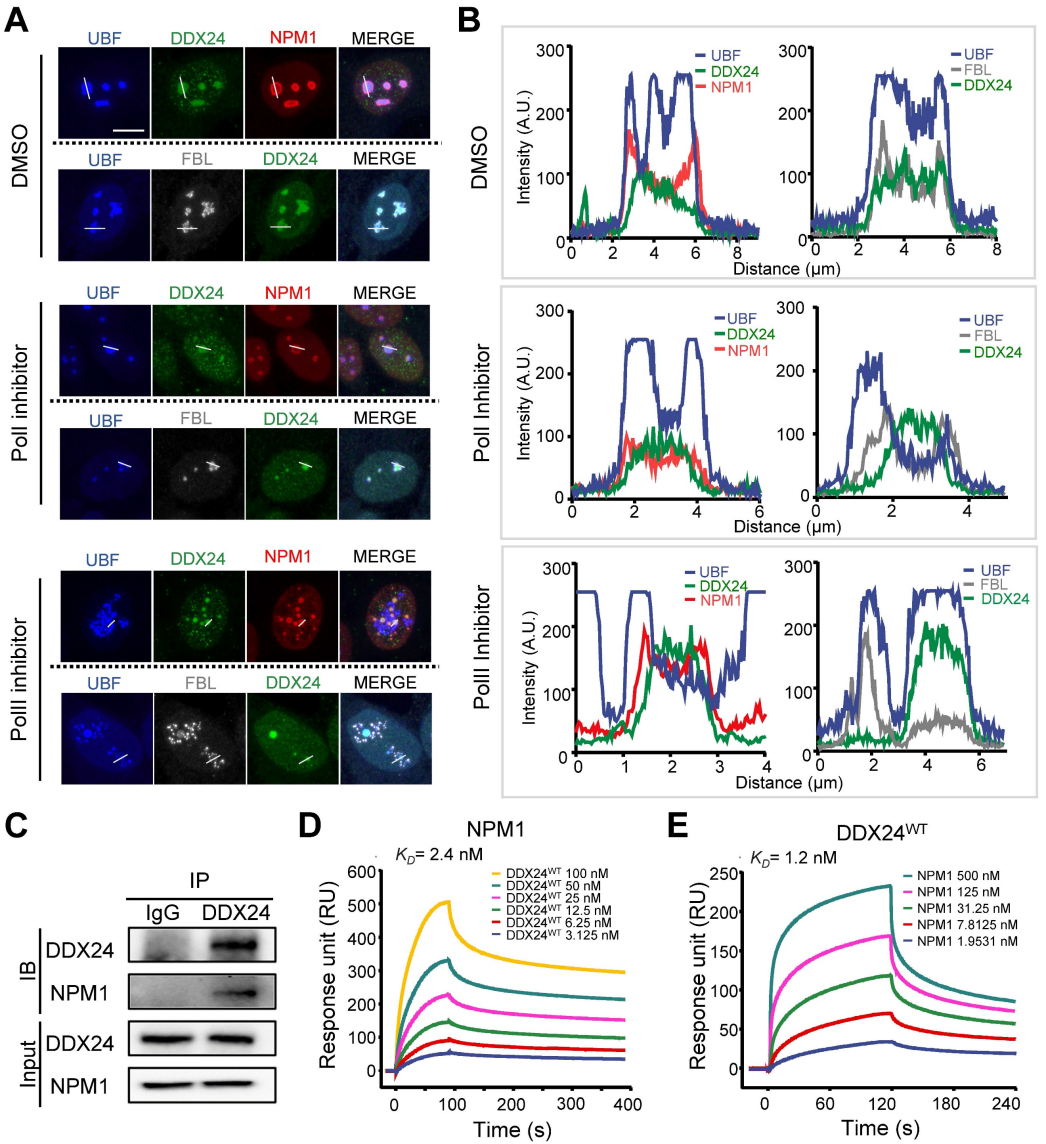
** DDX24 interacts with NPM1 in the granular component of the nucleolus**. **(A, B)** Representative confocal images of methanol-fixed HUVECs after 1-h incubation with DMSO, actinomycin-D (50 ng/ml) or flavopiridol (1 μM) (A) and plot profiles of while lines in (A) are shown in (B). Scale bar 50 μm. **(C)** Immunoblotting of DDX24 and NPM1 on HUVECs cell lysate immunoprecipitated with anti-DDX24 antibodies or control isotype-matched IgG. **(D, E)** Sensorgrams of DDX24^WT^ binding to NPM1 (D) and NPM1 binding to DDX24^WT^ (E). *K_D_* values were calculated as an average from 3 independent experiments. Images in (A, B) are representative of 2 independent experiments; Blots in (C) are representative of data obtained from 2 biological replicates.

Nucleolar proteins are distributed in different compartments in the nucleolus to exert distinct functions. Unlike FBL and UBF (the marker for DFC and FC respectively) that formed bead-like structures in ECs, DDX24 distributed evenly inside the nucleolus (**Figure 3A-B upper panel**). To further scrutinize the subcellular localization of DDX24, we disturbed nucleolar structure by stressing HUVECs with low doses of actinomycin D (RNA polymerase I inhibitor) or flavopiridol (RNA polymerase II inhibitor), which led to 'nucleolar segregation'^30^, a condition in which the DFC segregated from the GC**.** In these conditions, both FBL and UBF were dislodged from the NPM1 shell while DDX24 remained inside (**Figure 3A-B middle and bottom panels**). We further examined the structural integrity of the nucleolus in cells upon DDX24 knockdown. Although the NPM1-marked nucleolus dispersed, the tripartite structure of the nucleolus stayed intact without alteration of NPM1 expression level (**Supplemental Figure 4A-B**). Knockdown of NPM1 yielded nucleolus shrunken into small droplets while preserving the expression level of DDX24 (**Supplemental Figure 4A-B**). Simultaneously, we investigated the localization of mutant DDX24 and discovered that both DDX24^E271K^ and DDX24^WT^ were encompassed within the nucleolar NPM1 shell (**Supplemental Figure 5A-B**). Therefore, DDX24 appears to be one of the many proteins that are contained within the macromolecular network of the GC.

To characterize the role of DDX24 in the GC, we investigated its interaction with the scaffold protein NPM1 by co-immunoprecipitation (Co-IP) assay and proved their direct association (**Figure 3C**). Surface plasmon resonance (SPR) analysis further validated the affinity between these two proteins at the sub-nanomolar level (**Figure 3D-E; Supplemental Table 1**), yet the mutation of DDX24 barely affected its affinity with NPM1 (**Supplemental Figure 5C-D; Supplemental Table 1**). Taken together, our results elucidate that DDX24 is predominantly recruited to the GC of the nucleolus through association with the scaffold protein NPM1.

### Less DDX24^E271K^ partitions into NPM1/rRNA droplets

**Figure 4:**
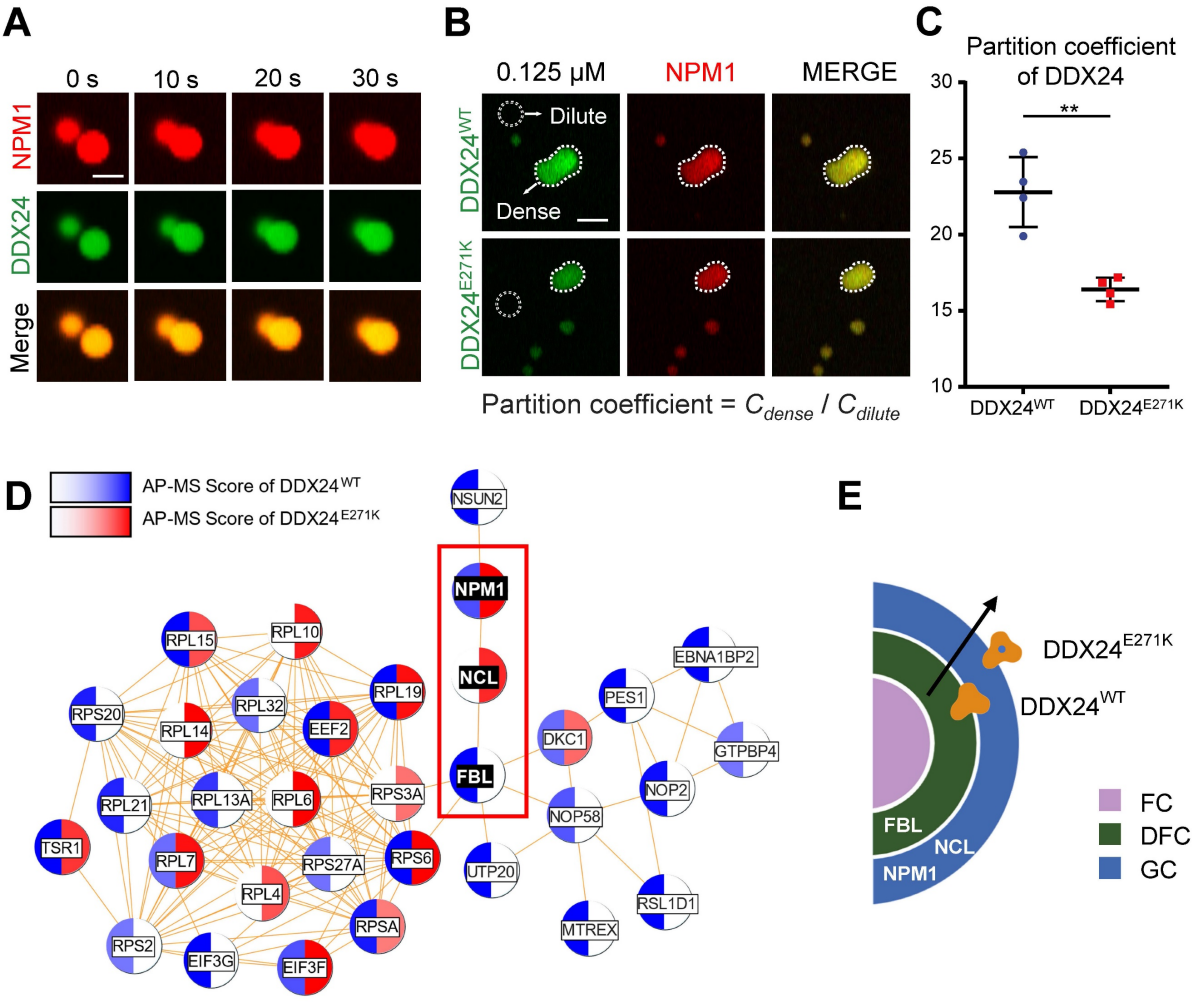
** DDX24 forms heterotypic droplets with NPM1 and rRNA. (A)** Representative confocal images of fusion event of phase-separated structures formed by 8 μM DDX24^WT^ protein (Alexa Fluor 488), 20 μM NPM1 (Alexa Fluor 594) and 100 μg/mL rRNA in LLPS buffer (20 mM Tris, 150 mM NaCl, 1 mM TCEP, pH 8.0). Scale bar 5 μm. **(B, C)** Representative confocal images of 0.125 μM DDX24^WT^ protein (Alexa Fluor 488) or 0.125 μM DDX24^E271K^ protein (Alexa Fluor 488) droplet formation with NPM1 (20 μM) and rRNA (100 μg/mL) in the same buffer as above (B) and the quantification of partition coefficient for the two DDX24 constructs (C), dense phase and dilute phase outlined by dotted line. Scale bar 10 μm. **(D)** Network analysis of DDX24^WT^ and DDX24^E271K^ associated proteins, protein-protein interaction data were retrieved from STRING database, AP-MS score was calculated from normalized spectral counts obtained from MS. **(E)** Schematic illustrating that the E271K mutation affects the subnuclear localization of DDX24. Scale bars, as indicated. Data are shown as mean values ± s.d. Images in (A, B) are representative of 2 independent experiments; *n* ≥ 4 HPFs for each group pooled from 2 independent experiments (C); *n* = 2 independent experiments (D). Two-tailed Student's t-test for (C). NS, not significant; **P≤ 0.01.

To further investigate the phase behavior of DDX24 in multicomponent condensates mimicking nucleolus, we adopted a well-established in vitro reconstitution system of GC^12^,^31^,^32^. As expected, NPM1 (20 μM), DDX24 (10 μM) and rRNA (100 μg/mL) formed condensates in a Tris-NaCl LLPS buffer (20 mM Tris, 150 mM NaCl, 1 mM TCEP, pH 8.0), which exhibited typical liquid droplet characteristics as demonstrated by droplet fusion (**Figure 4A**). Subsequently, we drew a 2D phase diagram titrating both NPM1 and DDX24 by monitoring droplets formation and turbidity. Similar to SURF6 and other non-ribosomal proteins as previously described^11^,^31^, both wildtype and mutant DDX24 lowered the critical concentration of NPM1 for LLPS (**Supplemental Figure 6A**), yet the differences made by the two constructs were negligible (**Supplemental Figure 6B**).

We proceeded to examine whether the mutation could affect the partitioning of DDX24 in physiological conditions by firstly measuring the protein concentration of endogenous DDX24 in HUVECs with quantitative Western blotting (**Supplemental Figure 7A-B**). Remarkably, under the physiological concentration (125 nM for DDX24 and 20 μM for NPM1), DDX24^E271K^ partitioned less into the dense phase than DDX24^WT^ did (**Figure 4B-C**), complying with the discrepancy seen in the MOVLD patient tissues. To better specify the mutational effect of DDX24^E271K^ in vivo, we conducted Co-IP experiments followed by mass spectrometry (MS) analysis. We found that FBL was more abundant in DDX24^WT^-associated proteins while NCL (another marker for GC of the nucleolus) was more associated with DDX24^E271K^ (**Figure 4D-E**). The shift in potential interactions with markers of nucleolar compartments confirmed the difference in phase properties yielded by DDX24^E271K^ mutation. These results demonstrate that DDX24 can form multicomponent liquid droplets with NPM1 and rRNA in vitro, and DDX24^E271K^ exhibits reduced partitioning into the droplets.

### DDX24^E271K^ reduces the liquidity of NPM1 in biological condensates

**Figure 5:**
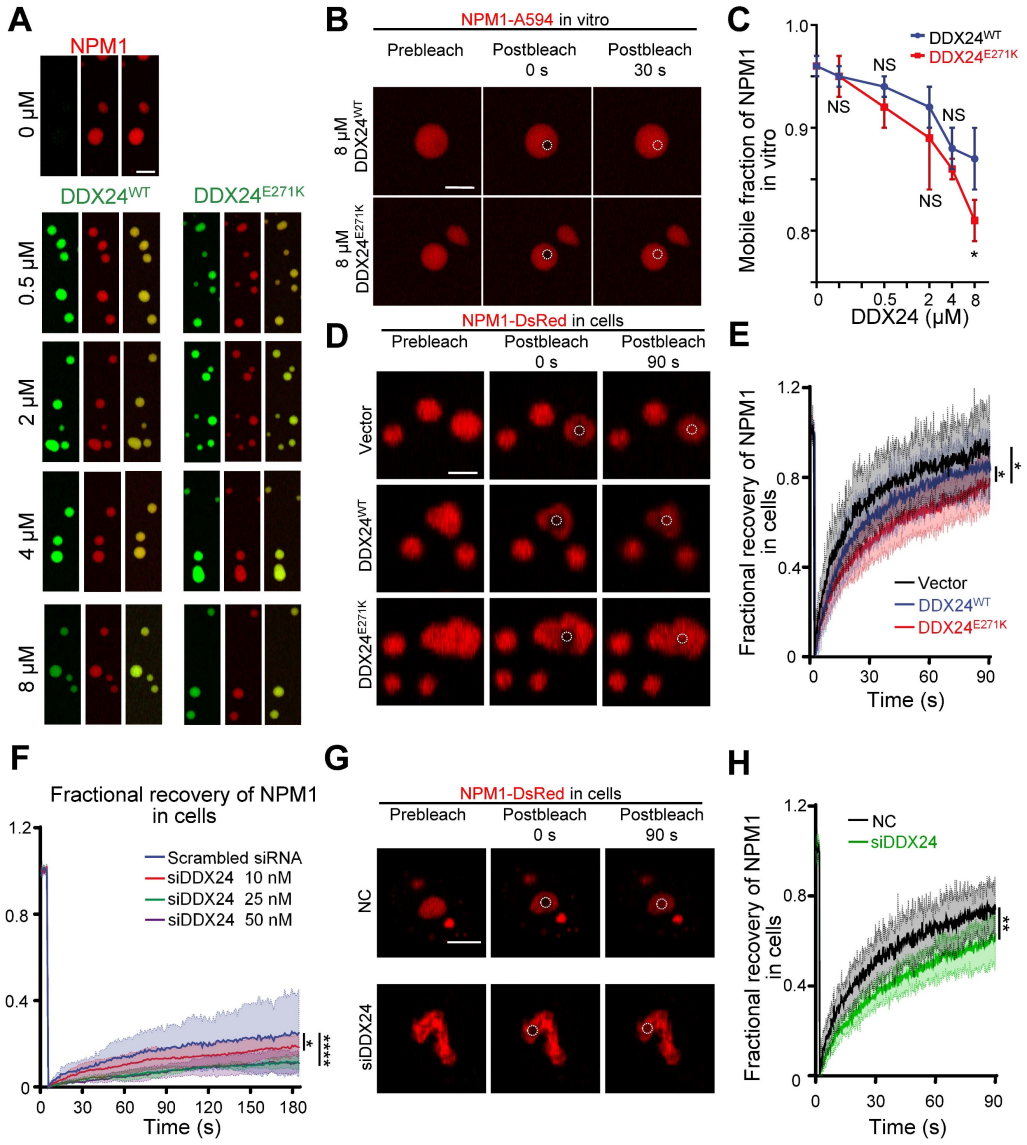
** DDX24 knockdown or DDX24^E271K^ reduces the liquidity of NPM1 in the nucleolus. (A)** Representative confocal microscopy images of droplets formed by 20 μM NPM1 (Alexa Fluor 594), 100 μg/mL rRNA and a titration series of DDX24^WT^ (Alexa Fluor 488) or DDX24^E271K^ (Alexa Fluor 488). **(B)** Representative confocal microscopy images of either 8 μM DDX24^WT^ (Alexa Fluor 488) or DDX24^E271K^ (Alexa Fluor 488) containing droplets in (A) before and after photobleached. **(C)** Change in mobile fraction of NPM1 throughout the titration series in (A) measured from FRAP curves. **(D, E)** Representative confocal images from live-cell NPM1-DsRed FRAP experiment in HUVECs transfected with DDX24 constructs as indicated (D) and the FRAP curve of these cells (E). **(F)** FRAP curves of NPM1-DsRed in 293T cells after titrated siRNA knock-down targeting DDX24. **(G, H)** Representative confocal images from live-cell NPM1-DsRed FRAP experiment in HUVECs transfected with siRNA targeting DDX24 or scramble control siRNA (G) and the FRAP curve of these cells (H). FRAP ROI = 1 µm circular area in the center of the selected nucleolus or droplet as outlined by dotted line. Scale bar 5 μm. Data are shown as mean values ± s.d. Images in (A, B, D, G) are representative of 2 independent experiments; *n* ≥ 3 droplets for each group in (C); *n* ≥ 3 cells for each group in (E, F, H). Welch's t-test with Bonferroni-Dunn's multiple comparisons test for (C); Ordinary one-way ANOVA with Dunnett's multiple comparisons test for (E, F); Two-tailed Student's t-test for (H). NS, not significant; *P≤ 0.05, **P≤ 0.01, ****P≤ 0.0001.

Non-ribosomal nucleolar proteins can modulate the interaction network within the GC as clients and fine-tune the nucleolar material state^13^,^14^,^31^. To further explore the compositional change of droplets formed by the two constructs with NPM1/rRNA, we titrated DDX24^WT^ or DDX24^E271K^ with a constant concentration of NPM1 (20 μM) and rRNA (100 μg/mL) (**Figure 5A**). Nevertheless, the partition coefficient of either NPM1 or DDX24 differed little between the two constructs at physiologically irrelevant concentrations (**Supplemental Figure 8A-B**), indicating that DDX24^E271K^ did not alter the overall architecture of the in vitro DDX24/NPM1/rRNA droplet.

However, FRAP analysis showed that NPM1 molecules were less mobile in the presence of DDX24^E271K^ in contrast to DDX24^WT^ throughout the series (**Figure 5B-C, Supplemental Figure 8C**) and the difference became most prominent at 8 μM, yet the mobility of mutated DDX24 was comparable to that of wildtype (**Supplemental Figure 8D**). In consistent with the findings in vitro, the mobility of NPM1 molecules in the nucleoli of HUVECs overexpressing DDX24^E271K^ dropped significantly compared to those transfected with empty vector or DDX24^WT^ (**Figure 5D-E**).

To evaluate the phase behavior of NPM1 inside the nucleoli with DDX24 deficiency, we performed titrated knockdown of DDX24. Interestingly, live-cell FRAP results showed that the dynamics of NPM1 molecules in the nucleolus became less mobile when the concentration of WT DDX24 protein is decreased (**Figure 5F**). Similar results were obtained in HUVECs upon DDX24 knockdown (**Figure 5G-H**). Thus, our results indicate that either DDX24 mutation or depletion can reduce the liquidity of NPM1in biological condensates formed in vitro and in the nucleoli of living cells.

### DDX24^E271K^ impairs ribosome biogenesis and EC functions

**Figure 6:**
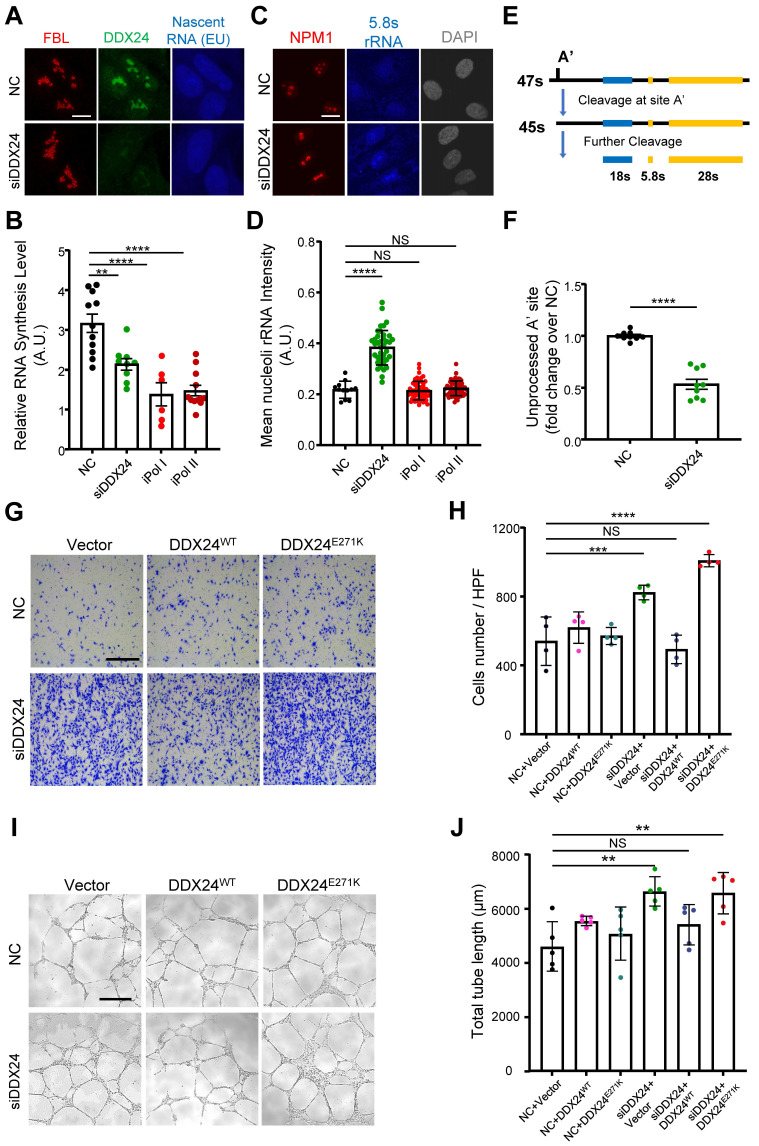
** DDX24 maintains nucleolar homeostasis and proper functions of endothelial cells. (A, B)** Representative confocal images of HUVECs transfected with control or siDDX24 siRNA (A) and quantification of nascent RNA synthesis following different stress treatment as indicated (B). Scale bar 10 μm. **(C, D)** Representative confocal images of NPM1 (Red) and 5.8s rRNA (blue) in HUVECs transfected with control or siDDX24 siRNA (C) and quantification of mean nucleoli intensity of 5.8s rRNA after different stress treatment as indicated (D). Scale bar 20 μm. **(E)** Simplified schematic representations of pre-rRNA processing, with the processing/cleavage sites, A' as indicated. **(F)** qPCR analyses examining the processing of the 47s rRNA A′ site in HUVECs transfected by siRNA targeting DDX24 or scramble siRNA. The level of unprocessed A' site was normalized to the level of 7SK RNA. **(G, H)** Representative phase contrast images (G) and quantification of cells per high-power field (H) of migrated HUVECs in knock-down and re-expression assay. Scale bar 50 μm. **(I, J)** Representative phase contrast images (I) and quantification of total length of branches (J) of tube formation in knock-down and re-expression assay. Scale bars 200 μm. Data are shown as mean values ± s.d. Images in (A, C, G, I) are representative of 2 independent experiments; *n* ≥ 6 HPFs for each group pooled from 2 independent experiments (B); *n* ≥ 12 cells for each group pooled from 2 independent experiments (D); *n* = 9 pooled from 3 independent experiments (F); *n* = 4 HPFs for each group pooled from 2 independent experiments (H); *n* = 5 wells for each group pooled from 2 independent experiments (I). Ordinary one-way ANOVA with Dunnett's multiple comparison test was used except for (F), which used two tailed Student's t-test. NS, not significant; *P≤ 0.05, **P≤ 0.01, *** *P*≤ 0.001, **** *P* ≤ 0.0001.

Reduced liquidity of scaffold protein NPM1 caused by DDX24^E271K^ may have considerable phenotypic consequences for nucleoli and ultimately pathological alterations for cells. To investigate the pathological impact of DDX24^E271K^ on HUVECs, we inspected nascent RNA biogenesis in HUVECs with lowered nucleolar DDX24 level acquired by DDX24 knockdown, using Pol I or Pol II inhibitor-treated groups as positive control. As expected, less nucleolar DDX24 perturbed global RNA biogenesis in HUVECs, recapitulating the effects by RNA polymerase inhibitors (**Figure 6A-B** and **Supplemental Figure 9A**). Furthermore, we investigated the maturation of pre-60s subunit by detecting 5.8s rRNA. Immunofluorescence microscopy data revealed that 5.8s rRNA was accumulated in the nucleoli, suggesting defective maturation of the 60s subunit, with reduced nucleolar DDX24 (**Figure 6C-D** and **Supplemental Figure 9B**). However, over-expressing either DDX24^WT^ or DDX24^E271K^ did not affect the nucleolar intensity of 5.8s rRNA (**Supplemental Figure 9C-D**). We also designed specific primers targeting the cleavage of the A' site in 47s rRNA to reflect the changes in pre-rRNA processing and found that the unprocessed 47s transcript was largely reduced in DDX24-deficient HUVECs (**Figure 6E-F**), indicating that nucleolar DDX24 is involved in limiting the efficiency of 47s rRNA cleavage.

Previously, we reported that DDX24 knockdown led to elevated EC migration and tube formation, but caused little effect on cell growth^17^. Here we ectopically expressed DDX24^WT^ or DDX24^E271K^ in DDX24-deficient HUVECs to rescue the cellular phenotype. Consistent with the changes in NPM1 phase behavior noted above, DDX24^WT^ abrogated the promoted migration upon DDX24 knockdown while re-expression of DDX24^E271K^ hardly made any difference (**Figure 6G-H**). Similarly, tube formation mirrored the results of cell migration (**Figure 6I-J**). To be noted, knockdown of NPM1 in ECs also boosted cell migration (**Supplemental Figure 9E**). In summary, our data elucidate the essential role of DDX24 in maintaining nucleolar homeostasis in that reduced liquidity of nucleolar scaffold protein NPM1 either by mutation or reduced expression impairs ribosome biogenesis and ultimately the function of ECs.

## Discussion

In the current work, we revealed that DDX24 maintains nucleolar homeostasis as a client via interacting with its associated protein, NPM1. The mutation that affects DDX24 LLPS properties can transduce its effect to the phase behavior of NPM1 and disrupt RNA biogenesis, ultimately causing vascular malformations.

MOVLD syndrome is characterized by insidious onset, deep location of the lesions, and poor responsiveness to conventional interventional treatment, making both diagnosis and treatment challenging. A biomarker to detect the minor changes at the early stage and to monitor disease progression is essential for the therapeutic intervention of such disease. Recently, alterations of nucleolar protein structure and localization have emerged as promising biomarkers for viral infection^33^,^34^, cancer^35^, and aging^36^. In neurodegenerative disease, for instance, nucleolar swelling and translocation of nucleolar proteins are observed in patient tissues and mouse models, and associated with Parkinson's disease and Alzheimer's disease^37^,^38^. To our knowledge, no report has related nucleolar morphology to vascular malformation so far. Our results highlight the nucleolar morphological change indicated by ruffled NPM1 staining and/or a lowered nucleolar DDX24 concentration in ECs as the potential biomarker for MOVLD syndrome.

Generally, dysfunctions of ribosome biogenesis, termed ribosomopathies, are caused by mutations in ribosomal assembly factors or r-proteins^39^. Many studies have proven that defects in ribosome biogenesis can cause accumulation of 5s RNP, which binds to Hdm2 and triggers activation of p53 signaling pathway^40^,^41^. Nevertheless, recent studies have suggested that the liquid-like properties of nucleoli are important for the assembly and exit of ribosomal subunits^11^,^32^. Ribosomopathies, in this regard, could potentially be caused by altered nucleolar material properties. Even though vascular malformation has never been viewed as or related to ribosomopathies, the importance of ribosome biogenesis in angiogenesis has been recently asserted. DDX21, another DEAD-box protein, has been found to contribute to the development of lymphatic vessels by balancing ribosome biogenesis and p53 response^42^. Our data unveil that reduced liquidity of NPM1 inside nucleoli caused by DDX24^E271K^ can hamper rRNA biogenesis and interrupt the assembly of ribosomal large subunits causing 5.8s rRNA accumulation in the nucleoli. In addition, the downstream effect of altered NPM1 phase behavior may converge on the p53 signaling pathway, as DDX24 has been identified as an activator of p53^43^, to influence cellular functions. Our work adds to the knowledge of how nucleolar phase behavior can be tuned by a nucleolar client (DDX24 in this case), and ultimately dictates the nucleolar homeostasis.

One-third of DEAD-box helicases in their family contain IDRs that are crucial in mediating LLPS to compartmentalize various RNA-processing reactions, exemplified by DDX3X and DDX4^18^. However, DDX24 was not expected to phase separate as previously reported^18^. In fact, DDX24 is a unique member of its family in that it has an insertion region within the helicase core domain, and according to our protein structure and disorder prediction, the insertion is intrinsically disordered. DDX24^E271K^ happens to reside in the IDR, a mutation altering a negative charged residue to a positive one, which may affect its interaction with RNAs or proteins, leading to the alteration in phase behavior of NPM1 and ribosomal dysfunction. Our data demonstrate that even though DDX24 alone is unlikely to phase separate at its physiological concentration in cells, it can be recruited to the NPM1/rRNA droplets and preserve a high level of partition. Mutations in IDRs may affect the general phase properties of the biomolecular condensates, inducing dysfunction of phase separation and mechanistically contributing to diseases^44^. For instance, mutations in the prion-like domain of FUS protein accelerate the conversion of dynamic FUS droplets to solid aggregates, leading to the early onset of ALS^45^. Likewise, we discover here that DDX24^E271K^ in the IDR perturbs DDX24 partitioning into the NPM1/rRNA droplets and gives rise to aberrant NPM1 phase behavior. After all, our work emphasizes the importance to measure the impact of genetic mutation concerning IDR on phase separation during phenotypic interpretation, especially the mutation in nucleolar proteins of which LLPS properties are essential to its homeostasis.

As the role of biomolecular condensates in mediating cellular function is increasingly revealed, new therapeutic approaches targeting aberrant phase separation have emerged. Mitrea et.al. proposed the concept of condensate-modifying therapeutics (c-mod) that peptides, small molecular drugs, or nucleic acids can affect the biophysical properties, condensates to treat diseases^46^. For example, avrainvillamide, a natural small molecular drug, could covalently bind to mutant NPM1 in acute myeloid leukemia (AML) restores nucleolar localization of the mutant, exhibiting promising therapeutic potential^47^. In this perspective, modulation of nucleolar material properties, either by gene editing or small-molecular drugs, may provide a novel strategy on the therapeutic intervention of diseases like vascular malformations.

## Materials and Methods

### Cell culture and materials

Human umbilical vein endothelial cells (HUVECs) were obtained from ScienCell (Catalog #8000), cultured in ECM (ScienCell, Catalog#1001) supplemented with 10% fetal bovine serum. HEK 293T cells were thawed from nitrogen stock from our lab, cultured in Dulbecco's modified Eagle's medium (Gibco) supplemented with 10% fetal bovine serum, 50 U/mL penicillin, and 50 mg/mL streptomycin (Gibco, Catalog#15140-122), HUVECs were used between passages 2 and 6 for experiments. Refresh medium every other day, dissociated for sub-culturing with TrypLE Select (Gibco, Catalog#12563011) when full. Gene knock-down of DDX24 was accomplished using predesigned and validated siRNA (Ribobio), transfected with Lipofectamine 3000 (Invirtrogen), and effective knock-down was confirmed by immunoblot analysis. For transfections with plasmids encoding DDX24^WT^-EGFP, DDX24^E271K^-EGFP and NPM1-DsRed, 70% confluent cells were transfected with 1 µg of plasmid per well of a six-well plate using Lipofectamine 3000. Overexpression in knockdown and re-expression assay was done by a lentiviral shuttle plasmid for DDX24^WT^ and DDX24^271^ (GeneChem), confirmed by immunoblot analysis. Cells were starved with low serum medium for 4 h before treatment of the following chemicals, Actinomycin D (APExBio, Catalog# A4448), Flavopiridol (MCE, Catalog# HY-10005). All chemicals were dissolved in DMSO and were diluted to make 1000X solution before adding to complete medium for treatments.

### RNA extraction and qPCR

Total RNA extraction was performed from cells using the FastPure Cell/Tissue Total RNA Isolation Kit (Vazyme, Catalog#RC111). RT-qPCR was performed using HiScript II One Step qRT-PCR SYBR Green Kit (Vazyme, CataLog# Q221). qPCR was performed in triplicate with a CFX96TM real-time system. Thermocycling conditions were 95 °C for 3 min, followed by 40 cycles of 95 °C for 15 s, 60 °C for 45 s. Gene expression was normalized to the constitutively expressed housekeeping gene 18S rRNA or GADPH, and relative expression was calculated and plotted using the ΔΔCt method.

The following primers were used to assess gene expression: A' rRNA-forward 5'-TGTCAGGCGTTCTCGTCTC-3'; A' rRNA-reverse 5'-AGCACGACGTCACCACATC-3'; 7SK snRNA-forward 5'-GTCTTCGGTCAAGGGTATACGAG-3'; 7SK snRNA-reverse 5'-CGCCTCATTTGGATGTGTC-3'; DDX24 mRNA-forward 5'-CAGTTTGCCCGTCTGGAAGA-3'; DDX24 mRNA-reverse 5'-ACGTGGGACCTGGTAATGGA-3'; 45S-pre-rRNA forward GAACGGTGGTGTGTCGTTC; 45S-pre-rRNA reverse GCGTCTCGTCTCGTCTCACT; 28S rRNA forward CAGGGGAATCCGACTGTTTA; 28S rRNA reverse ATGACGAGGCATTTGGCTAC; 18S rRNA forward AAACGGCTACCACATCCAAG; 18S rRNA reverse CCTCCAATGGATCCTCGTTA; 5.8S rRNA forward CTCTTAGCGGTGGATCACTC; 5.8S rRNA reverse GACGCTCAGACAGGCGTAG; β-actin forward GCTCGTCGTCGACAACGGCTC; β-actin reverse CAAACATGATCTGGGTCATCTTCTC.

### Co-immunoprecipitation and western blotting

Endogenous DDX24 pulldown assays were performed with anti-DDX24 antibody (Bethyl, Catalog # A300-698A) using Pierce™ Co-Immunoprecipitation Kit (Thermo, Catalog # 26149) by following the manual instructions. Normal rabbit IgG (Santa Cruz) served as a control. The pull-down proteins were resolved via SDS-PAGE and silver stained followed by mass spectrometry (MS) identification (FitGene Biotechnology, China). The AP-MS (affinity purification - mass spectrometry) scores were obtained following the integrated pipeline of REPRINT.

For all western blotting analysis, protein lysates from cells or fresh frozen tissues and co-immunoprecipitation complexes were resolved on a precast 4-12% gradient gel (Genscript), with appropriate primary antibodies recognized DDX24 (Bethyl, Catalog # A300-698A), NPM1 (Invitrogen, Catalog # 32-5200), β-Actin (Proteintech, Catalog # 60008).

### Nascent RNA imaging

Cell-Light EU Apollo-488 RNA Imaging Kit (Ribobio, Catalog# C10316-3) was used. Cells were seeded in 15 mm glass-bottom confocal dishes at 10,000 cells per dish. Twenty-four hours later, cells were treated with chemicals and incubated with 0.5 mM ethyl uridine (EU) for 1 h. Cells were washed thoroughly and fixed after EU incubation. EU was detected using the imaging kit and the cells were stained with antibodies and other fixed-cells stains.

### Immunofluorescence microscopy

FFPE slides were deparaffinized and rehydrated, heat-induced antigen retrieval was performed with Tris/EDTA (pH=9) buffer for 30 min in water bath before antibody staining. Cells were seeded into 15 mm glass-bottom confocal dishes and fixed for 15 min in methanol at 4 ℃ before staining. Slides or dishes were then incubated in TBST (0.1% Triton X-100) for 10 min, blocked (10% Goat serum+ 1% BSA + 1% Glycine + 0.05% Tween) for 1h at room temperature before incubation of the first 2 primary antibody from rabbit and mouse overnight at 4 ℃. Alexafluor488 conjugated Goat anti-mouse Fab fragment and Alexafluor555 conjugated Goat anti-rabbit IgG were used to probe the first 2 target, the slides were then blocked with Goat anti-mouse Fab fragment. The third primary antibody from mouse was added to the slides to incubate overnight at 4 ℃. Alexafluor647 conjugated Goat anti-mouse IgG was used to probe the last target. The slides were mounted with DAPI-added antifade mounting medium before developing confocal image using Zeiss 880 confocal laser scanning microscope. The following primary antibodies were used: mouse anti-NPM1 (Invitrogen Catalog#32-5200, 1:200), rabbit anti-DDX24 (Sigma-Aldrich Catalog # HPA002554, 1:100), mouse anti-CD34 (Invitrogen Catalog # MA1-10202, 1:500), mouse anti-FBL (Invitrogen Catalog # MA3-16771, 1:200), mouse anti-UBF (Santa Cruz Catalog # sc-13125, 1:200), mouse anti-rRNA (Novus Catalog # NB100-662). Assessment of staining area and intensity was performed using custom pipeline in CellProfiler^48^. Intensity profiles across the cell nucleolus were plot in ImageJ^49^.

### Cellular function assays

Cell transmigration assay: HUVECs (50,000 cells/well) were plated on inserts (Transwell chambers 6.5 mm; Corning) with 8 μm pores and incubated in the upper chamber at at 37 °C in 5% CO2 for 6 h toward the lower chamber. Cells in the lower chamber were then stained with Crystal Violet (V5265) and counted in three random fields using an inverted microscope. Tube formation assay: HUVECs (10,000 cells/well) were plated on Matrigel (Corning, 354248) in 96-well plate at 37 °C in 5% CO2 for 8 h before image collection using an inverted microscope. Angiogenesis analysis was performed using a customized ImageJ plugin as previously reported^50^. Cell proliferation assay: HUVECs (5,000 cells/well) were plated in a 96-well plate and incubate at 37 °C in 5% CO2 for a specific length of time (4, 24, 48 or 72 h). Then 10 μL of CCK-8 solution was added to each well to incubate for another 2 h before absorbance measurement at 450 nm with a microplate reader.^50^.

### Cloning, protein expression and purification

For DDX24: Genes encoding human wildtype DDX24(residues from 28 to 859) were cloned in frame with an N-terminal 6x His-SUMO tag, followed by a ULP1 cleavage site. The DDX24 genes were subcloned into the pRSF-Duet-1 vector and expressed in the E. coli Rosetta strain. Cells from 10L culture were lysed by sonication in Buffer A [50 mM Tris, pH 8.0; 500 mM NaCl; 2 mM β-mercaptoethanol (βME)], supplemented with EDTA-free protease inhibitor (Targetmol). The soluble fraction of the lysate was loaded onto a Ni-NTA column and the bound protein eluted with a linear gradient of Buffer B [50 mM Tris, pH 8.0; 500 mM NaCl, 2 mM βME; 300 mM imidazole]. The 6× His-SUMO tag was removed by cleavage with ULP1 protease during overnight dialysis against 2 L of Buffer C [50 mM Tris, pH 8.0; 150 mM NaCl;2 mM βME;2 mM EDTA] at 4°C. The cleaved product loaded on another Ni-NTA affinity column for tag and protease removal. The flow-through soluble fraction were further loaded on a heparin column for nucleic acid removal and the bound protein eluted with a linear gradient of Buffer D [50 mM Tris, pH 8.0; 2000 mM NaCl, 2 mM βME; 2 mM EDTA]. The desire protein were finally purified via size-exclusion chromatography in buffer E [10 mM hepes, pH 7.5; 150 mM NaCl]. Fractions containing DDX24 or NPM1, confirmed by SDS-PAGE, were flash frozen and lyophilized.

For NPM1: Genes encoding human wildtype NPM1(residues from 1 to 290) were cloned in frame with a 6× His-SUMO tag followed by a ULP1 cleavage site. Genes were subcloned into the pRSF-Duet-1 vector and expressed in the E. coli Rosetta strain. All recombinant NPM1 proteins were expressed in E. coli Rosetta strain in Terrific Broth media in the presence of 50 mg/L Kanamycin. The bacterial cultures were grown at 37 °C to an optical density OD 0.6 ~ 0.8. Protein expression was induced by the addition of 0.1mM IPTG, the temperature was lowered to 16 °C and the cultures incubated overnight. Bacterial cultures were harvested by centrifugation and lysed in buffer A [50 mM Tris, pH 8.0; 500 mM NaCl; 2 mM β-mercaptoethanol (βME)], by sonication on ice. The proteins were purified from the soluble fraction, by passing through a Ni-NTA affinity column and eluting with a linear gradient of buffer A containing 300 mM Imidazole. The affinity tags were removed in an overnight dialysis step at 4 °C, against 2 L 50 mM Tris pH 8.0, 150 mM NaCl, 2 mM EDTA buffer, in the presence of ULP1 protease. The cleaved proteins were loaded on a Ni-NTA affinity column for tag and protease removal. The flow-through soluble fraction were further loaded on a heparin column for nucleic acid removal and the bound protein eluted with a linear gradient of Buffer D [50 mM Tris, pH 8.0; 2000 mM NaCl, 2 mM βME; 2 mM EDTA]. The desire protein were finally purified via size-exclusion chromatography in buffer E[10 mM hepes, pH 7.5; 150 mM NaCl] .The fractions containing the protein of interest were lyophilized. Lyophilized protein was resuspended in buffer containing 6 M guanidinium hydrochloride to a final monomer concentration of 100 µM and refolded by dialysis against three changes of 1 L 10 mM hepes, 150 mM NaCl, pH 7.5, at 4 °C.

### Surface plasmon resonance (SPR) Assay

Biacore T200 instruments (GE Healthcare) were used to evaluate the binding affinity of compounds to DDX24 or NPM1 via SPR, as previously described^51^. Briefly, DDX24 or NPM1 was immobilized on the surface of CM5 chip by using amine-coupling approach at a flow rate of 10 μL/min in 10 mM sodium acetate buffer (pH 5.5). The sensor surface was activated with a 7 min injection of the mixture of 50 mM N-hydroxy-succinimide (NHS) and 200 mM 1-ethyl-3-(3-dimethylaminopropyl) carbodiimide (EDC). Then 100 μg/mL of NPM1 or DDX24 was injected to reach the target level of 5000 RU and the surface was blocked with 1 M ethanolamine, pH 8.5. Series concentrations of compounds were injected into the flow system and analyzed for 90 s, and the dissociation was 120 s. All binding analysis was performed in phosphate buffered saline (PBS) with 0.05% (v/v) Tween-20 and 1% DMSO, pH 7.4, at 25 °C. Prior to analysis, double reference subtractions and solvent corrections were made to eliminate bulk refractive index changes, injection noise, and data drift. The binding affinity was determined by fitting to a Langmuir 1:1 binding model within the Biacore Evaluation software (GE Healthcare).

### Fluorescent labeling of purified protein

NPM1 were N-terminally labeled using Alexa Fluor 594 succinimidyl ester dye conjugates (Invitrogen, Catalog#A37565), DDX24 were N-terminally labeled using Alexa Fluor 488 succinimidyl ester dye conjugates (Invitrogen, Catalog# A20100) following the manufacturer's protocol. Protein concentration and labeling efficiency were determined according to manufacturer's protocol. 10% fluorescently labeled proteins were mixed with 90% unlabeled. NPM1 constructs were dialyzed, ratio adjusted, and folded as described previously. All labeled proteins were lyophilized and reconstituted in Buffer E described above before microscopy investigation.

### In vitro liquid-liquid phase separation assay

For unlabeled LLPS assay, to induce phase separation at low salt condition, the purified stock solution of NPM1, DDX24^WT^ and DDX24^E271K^ were diluted in corresponding buffer as indicated to needed concentration in a total volume of 20 μl. 2 μl of each sample was pipetted onto a glass slide and imaged using an inverted microscope (Leica). The rest of each sample was submitted to turbidity assessment. For fluorescent LLPS assay, purified stock solution of of NPM1 (10% NPM1-A594), DDX24^WT^ (10% DDX24^WT^-A488) and DDX24^E271K^ (10% DDX24^E271K^-A488) were diluted in corresponding buffer as indicated to needed concentration in a total volume of 10 μl and incubated for 5 min prior to analysis. Each sample was pipetted onto a glass slide and imaged using Zeiss 880 confocal laser scanning microscope with a 63× oil objective. The partition coefficient was defined and determined as previously described^12^. All analyses were performed using ImageJ.

### Fluorescence recovery after photobleaching (FRAP)

FRAP assay was conducted using the FRAP module of Zeiss 880 confocal laser scanning microscope. A circular region of interest (ROI) of 1 μm in diameter was photobleached using an appropriate laser beam and time-lapse images were collected. Fluorescence intensity was measured using ImageJ, and FRAP curve fitting were performed using standalone version of EasyFRAP^52^. T-half (half maximal recovery time) from curve fitting were selected for statistical analyses.

### Turbidity assays

Phase separation of NPM1 and DDX24 was determined by monitoring solution turbidity in the presence of rRNA (100 μg/mL) in 20 mM Tris, 150 mM NaCl, 1 mM TCEP, pH 8.0. To construct the phase diagram for NPM1-DDX24 solutions, turbidity was monitored over a wide range of NPM1 and DDX24 concentrations. Samples were incubated for 10 min at room temperature and vortexed prior to measuring the absorbance at 340 nm using a NanoDrop 2000c spectrophotometer (Thermo Scientific, Waltham, MA, USA). Measurements were performed in triplicate. Solutions were scored positive for LLPS when A340 ≥ 0.1.

### Statistics

Statistical analyses were performed using GraphPad Prism 8. Unless otherwise indicated, when comparing two groups with normal distribution, a two-tailed Student's t-test was used, if non-normally distributed, two-tailed Mann-Whitney test was used. Unless otherwise indicated, for multiple group comparisons, one-way analysis of variance (ANOVA) test was used for normally distributed data, Kruskal-Wallis test was used for non-normally distributed. Normal distribution was assessed by Shapiro-Wilk test. All error bars represent the standard deviation of the mean (s.d.) unless otherwise indicated.

## Supplementary Material

Supplementary figures and table.
