# Supplementary material for: DDX24 Mutation Alters NPM1 Phase Behavior and Disrupts Nucleolar Homeostasis in Vascular Malformations
Source: Int J Biol Sci. 2023 Aug 6;19(13):4123–38. doi: 10.7150/ijbs.84097 (PMC10496494; doi:10.7150/ijbs.84097)
Supplement: Supplementary file 1 — Supplementary figures and table. [file ijbsv19p4123s1.pdf]

SUPPLEMENTAL FIGURE LEGENDS

Fig. S1 Knockdown and re-expression assays performed on HUVECs. (A) Cell lysates of HUVECs in knockdown and re-expression assays immunoblotting with indicated antibodies. Blots are representative of data obtained from 2 biological replicates. **(B)** Quantification of the ratio of DDX24 nucleoli intensity / DDX24 nucleoplasm intensity from the knockdown and re-expression assay. **(C, D)** Quantification of the average size of nucleolus (C) and the number of nucleoli per nucleus (D) from the assay. $n > 100$ cells pooled from 2 independent experiments; data show the median, quartiles, and maximum/minimum values; Kruskal-Wallis test with Dunn's multiple comparisons test was used. NS, not significant; **** $P \leq 0.0001$.

Fig. S2 Sequence analysis and structure modeling. (A) AlphaFold model depicts DDX24 domains or regions with different confidence in various color as indicated. **(B)** Overall structure prediction of DDX24. Insertion region (IR) is shown in yellow, Glu 271 in red. **(C)** Analysis of intrinsically disordered domain of other DEAD-box helicase proteins. NTD, N-terminal domain; CTD, C-terminal domain.

Fig. S3 Phase properties of DDX24^{WT} and DDX24^{E271K} containing condensates in vitro and in HUVECs. (A) Representative images of 4 μM DDX24^{WT} protein (Alexa Fluor 488) or 4 μM DDX24^{E271K} protein (Alexa Fluor 488) condensate formation in the buffer condition as noted. Scale bar 50 μm. **(B)** Droplet turbidity curve of titration of DDX24^{WT} or DDX24^{E271K} into 100 μg/mL rRNA with or without the presence of PEG-400 (20%) in LLPS buffer (20 mM Tris, 150 mM NaCl, 1 mM TCEP, pH 8.0). **(C)** Droplet turbidity curve of 10 μM DDX24^{WT} protein or 10 μM DDX24^{E271K} protein in LLPS buffer (20 mM Tris, 1 mM TCEP, 100 μg/mL rRNA, pH 8.0) with NaCl (ranging from 50-500 mM). **(D, E)** Representative confocal images from live-cell DDX24 FRAP experiment in HUVECs transfected with DDX24-EGFP constructs as indicated (D) and the FRAP curve of these cells (E). Scale bar 5 μm. FRAP ROI = 1 μm circular area in the center of the selected nucleolus outlined by dotted line. Data are shown as mean values ± s.d.. Images in (A, D) are representative of 2 independent experiments; $n = 3$ independent experiments (B, C); $n \geq 3$ cells pooled from 2 independent experiments (E). Ordinary two-way ANOVA for (B); Welch's t-test with Bonferroni-Dunn's multiple comparisons test for (C); two-tailed Student's t-test for (E). NS, not significant; **** $P \leq 0.0001$.

Fig. S4 DDX24 and NPM1 knockdown experiment on HUVECs. (A) Cell lysates of HUVECs transfected with different siRNA targeting DDX24 or NPM1 immunoblotting with indicated antibodies. Blots are representative of data obtained from 2 biological replicates. **(B)** Representative confocal images of methanol-fixed HUVECs after transfection siRNA targeting DDX24 or NPM1. Scale bar 10 μm. Images are representative of 2 independent experiments.

Fig. S5 Difference in NPM1 affinity and nucleolar localization between the two DDX24 constructs. (A, B) Representative confocal images of HUVECs after transfection with DDX24^{WT}-FLAG or DDX24^{E271K}-FLAG (A), plot profiles of the dotted white lines in (A) are shown on the right (B). Scale bar 10 μm. Images are representative of 2 independent experiments. **(C, D)** Sensorgrams of DDX24^{E271K} binding to NPM1 (C) and NPM1 binding to DDX24^{E271K} (D). K_D values were calculated as an average from 2 independent experiments.

Fig. S6 Phase separation of NPM1/rRNA/DDX24 droplets (A) Phase diagram of DDX24^{WT} or DDX24^{E271K} (ranging from 2^{-9}-4 μM) in the same buffer as Fig. 4A with NPM1 (ranging from 0-100 μM) and 100 μg/mL rRNA. The formation of phase-separated structures was determined by bright field microscopy. **(B)** Droplet turbidity curve of titration of DDX24^{WT} or DDX24^{E271K} into 2.5 μM NPM1 and 100 μg/mL rRNA in the same buffer as above. Data are shown as mean values ± s.d.. n = 3 independent experiments (A, B). Ordinary two-way ANOVA was used for (B). NS, not significant.

Fig. S7 Endogenous concentration of DDX24 and NPM1 in HUVEC. (A, B) Quantification result of endogenous DDX24 (A) and NPM1 (B) protein concentration based on immunoblot densitometry analysis performed on cell lysates of HUVECs and purified protein.

Fig. S8 Partition coefficient of DDX24 and NPM1 throughout the titration assays. (A, B) Curves of partition coefficient of DDX24 (A) and NPM1 (B) throughout the titration of DDX24^{WT} (Alexa Fluor 488) or DDX24^{E271K} (Alexa Fluor 488) into 20 μM NPM1 and 100 μg/mL rRNA in LLPS buffer (20 mM Tris, 150 mM NaCl, 1 mM TCEP, pH 8.0). **(C)** FRAP recovery curves of NPM1 in droplets throughout the titration series in Fig. 5B. **(D)** FRAP recovery curves of DDX24^{WT} (Alexa Fluor 488) or DDX24^{E271K}

(Alexa Fluor 488) in droplets from the titration series in Fig. 5B at 8 μM. Data are shown as mean values ± s.d.. n = 3 experiments (A, B, C, D); Welch's t-test with Bonferroni-Dunn's multiple comparisons test for (A) and ordinary two-way ANOVA for (B); Two-tailed Student's t-test for (D). NS, not significant; ** $P \leq 0.01$.

Fig. S9 Colocalization and functional assays in HUVECs. (A) Representative confocal images of HUVECs after 1-h incubation with actinomycin-D (50 ng/ml) or flavopiridol (1 μM) after 30 mins incorporation of EU. Scale bar 10 μm. **(B)** Representative confocal images of NPM1 (Red) and 5.8s rRNA (blue) in HUVECs after 1-h incubation with actinomycin-D (50 ng/ml) or flavopiridol (1 μM). Scale bar 20 μm. **(C, D)** Representative confocal images of NPM1 (Red) and 5.8s rRNA (blue) in HUVECs transfected with N-terminal FLAG tagged $DDX24^{WT}$ or $DDX24^{E271K}$ (C) and quantification of mean nucleoli intensity of 5.8s rRNA (D). Scale bar 20 μm. **E**, Percentage of migrated cells in control and NPM1 knock-down HUVECs determined by DAPI staining after 8 h transwell incubation. Images in (A, B, C) are representative of 2 independent experiments; $n \geq 20$ cells for each group pooled from 2 independent experiments (D); $n \geq 10$ for each group pooled from 2 independent experiments (E). Ordinary one-way ANOVA with Dunnett's multiple comparison test was used in (D, E). NS, not significant; **** $P \leq 0.0001$.

Supplemental Table 1 SPR parameters calculated from SPR signal in Fig. 3D-E and Fig. S5C-D.

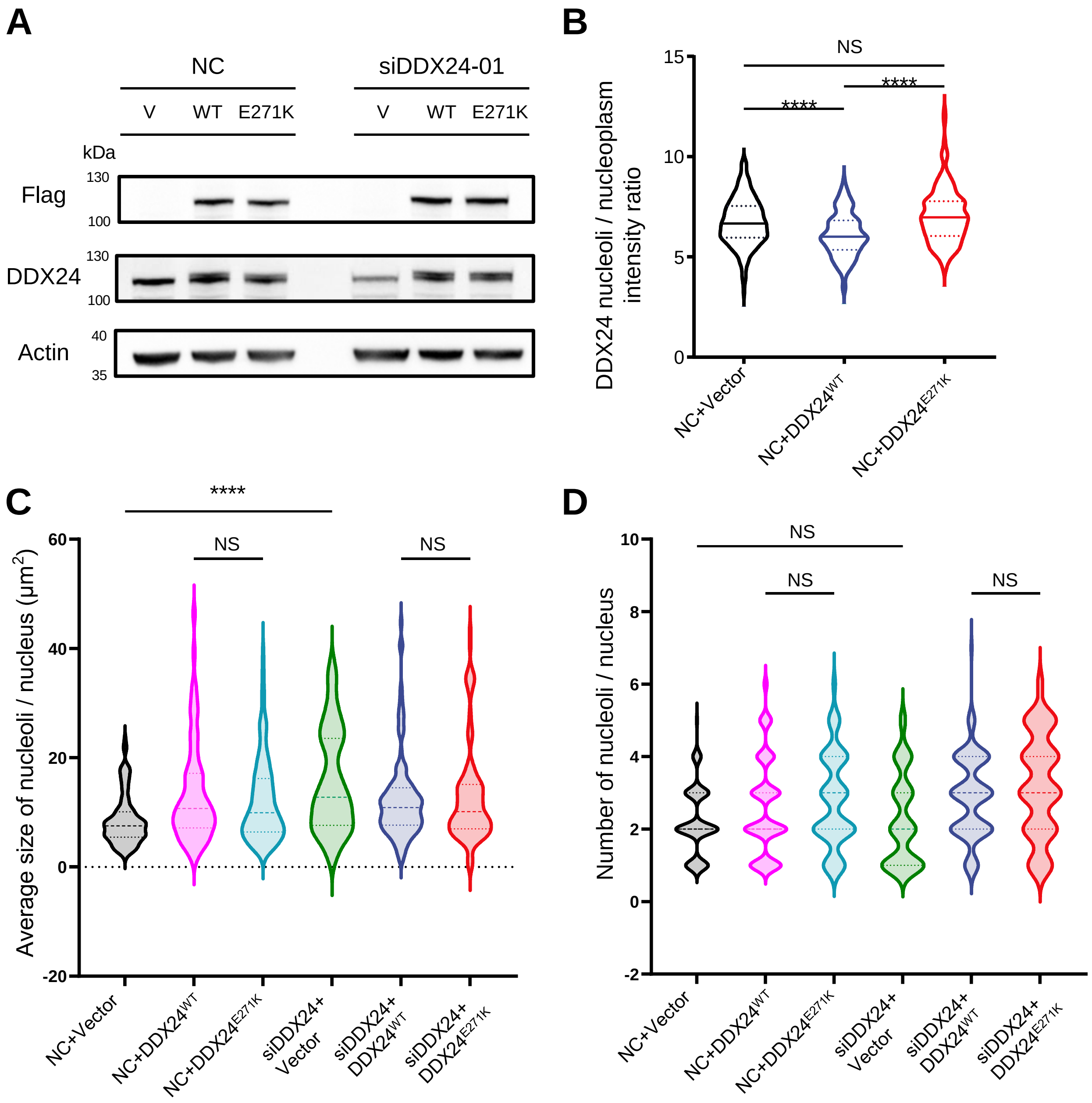

Supplemental Figure 1. Knockdown and re-expression assays performed on HUVECs.

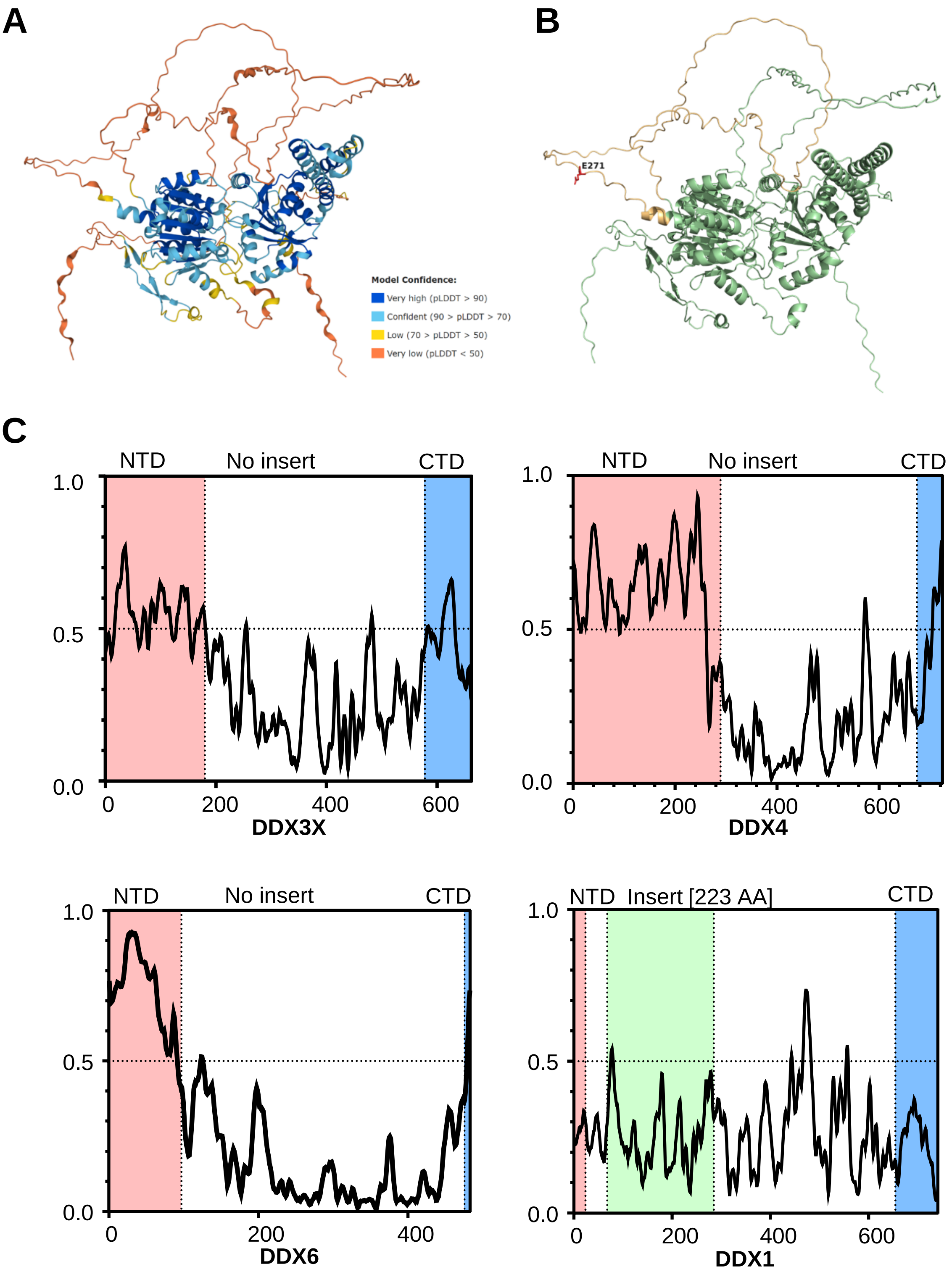

Supplemental Figure 2. Sequence analysis and structure modeling.

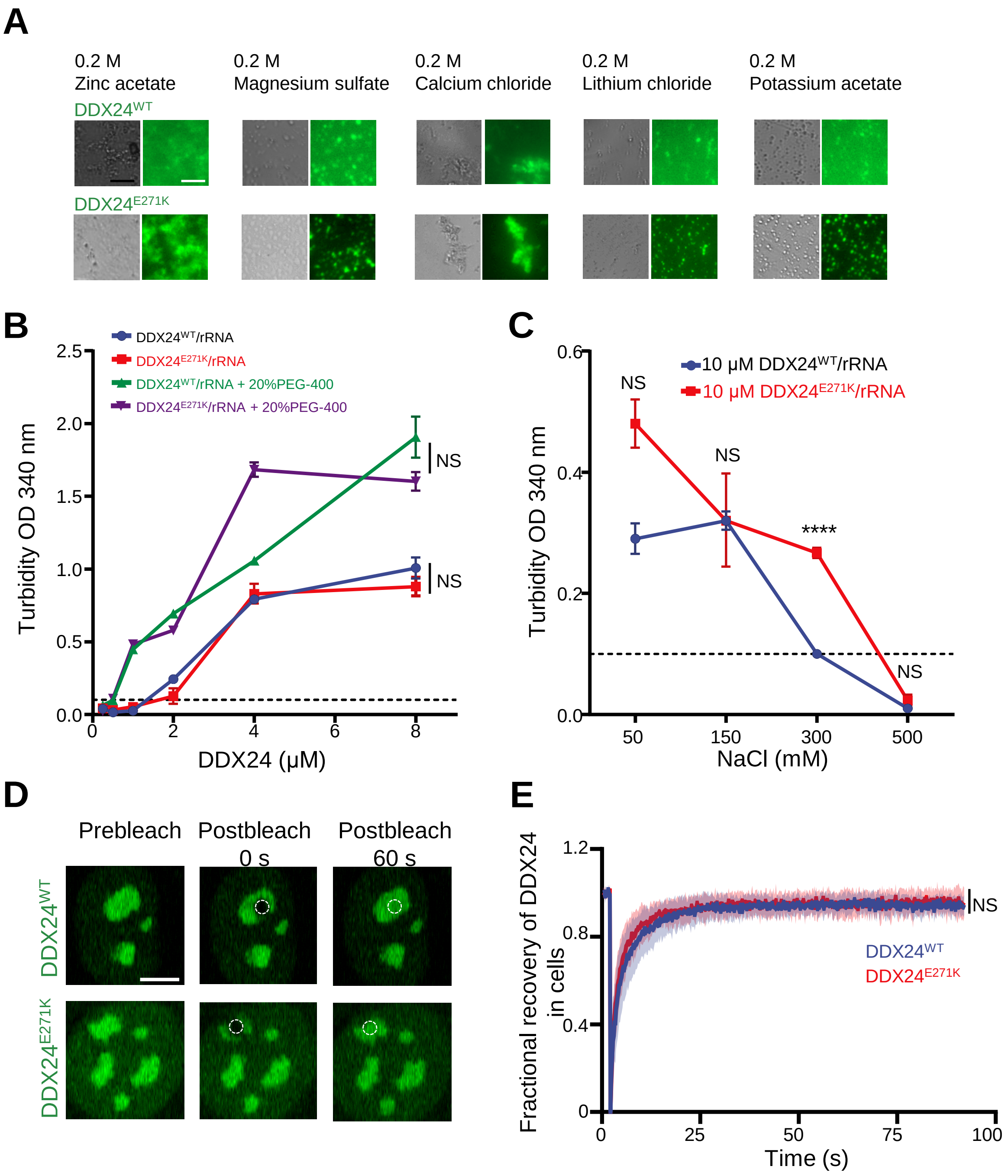

Supplemental Figure 3. Phase properties of DDX24^{WT} and DDX24^{E271K} containing condensates in vitro and in HUVECs.

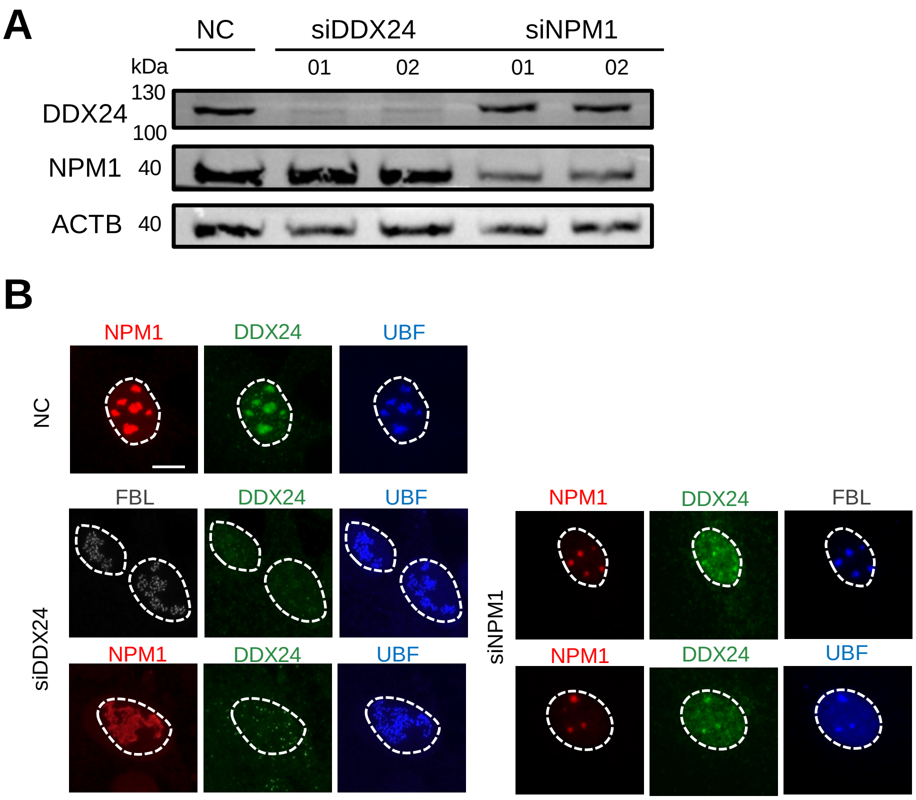

Supplemental Figure 4. DDX24 and NPM1 knockdown experiment on HUVECs.

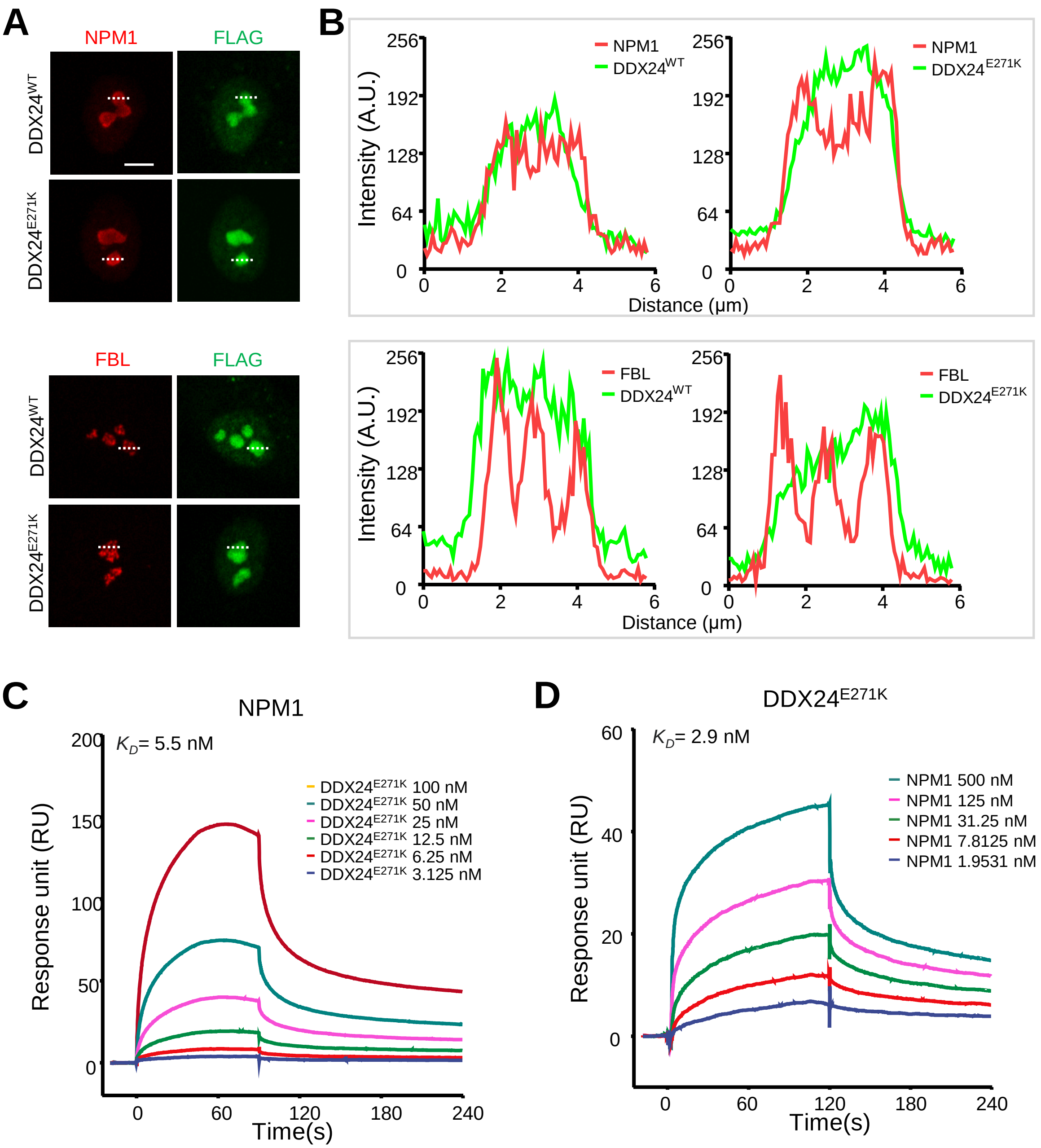

Supplemental Figure 5. Difference in NPM1 affinity and nucleolar localization between the two DDX24 constructs.

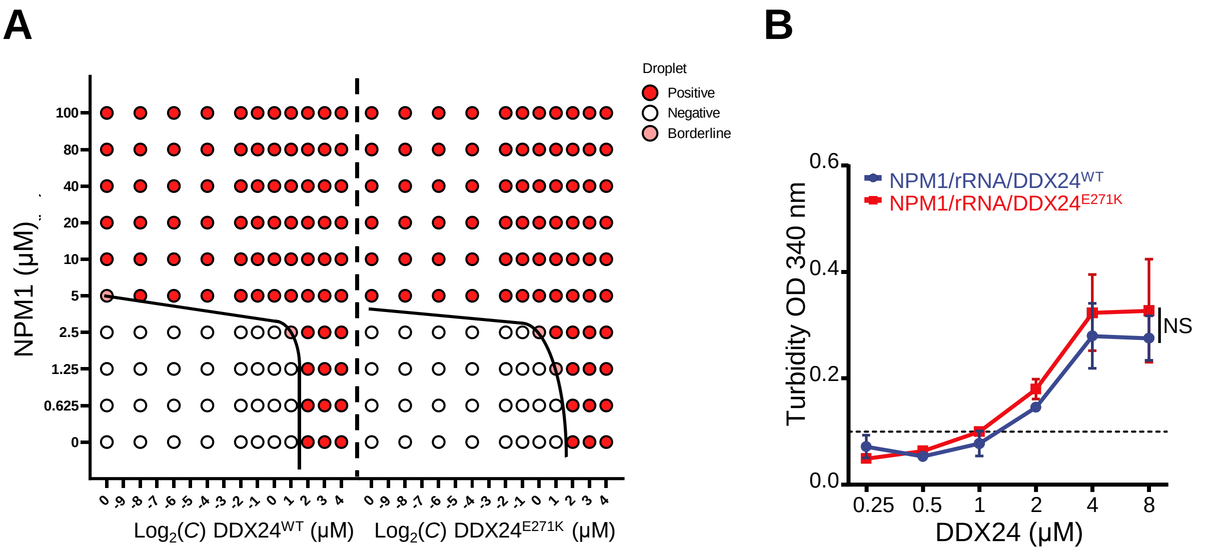

Supplemental Figure 6. Phase separation of NPM1/rRNA/DDX24 droplets.

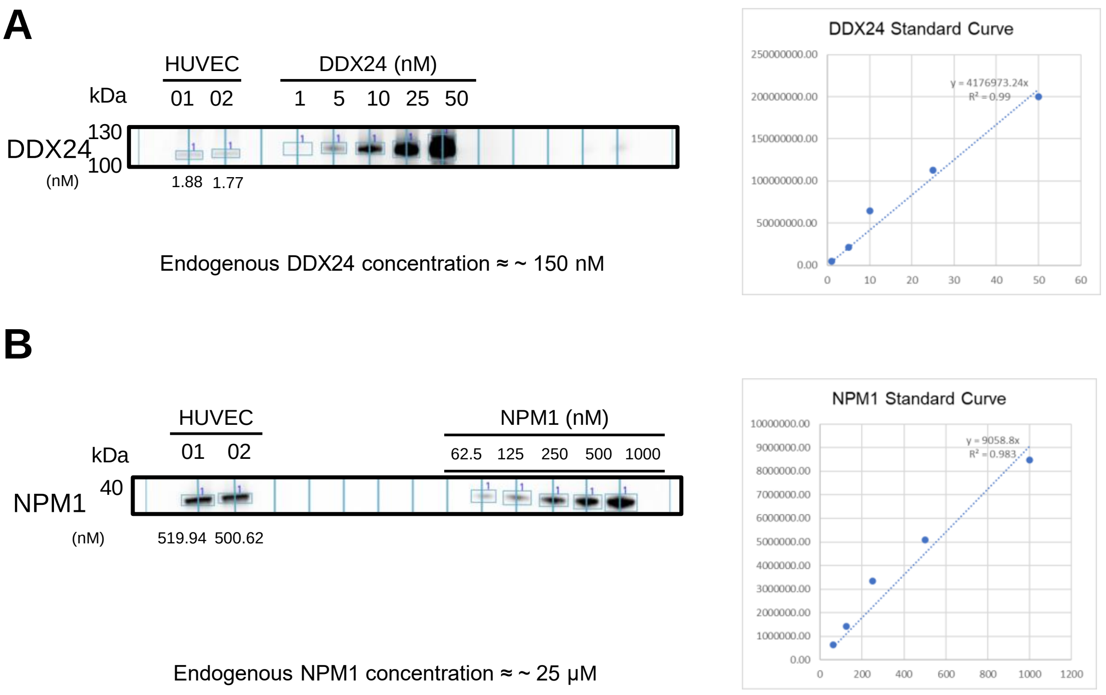

Supplemental Figure 7. Endogenous concentration of DDX24 and NPM1 in HUVECs.

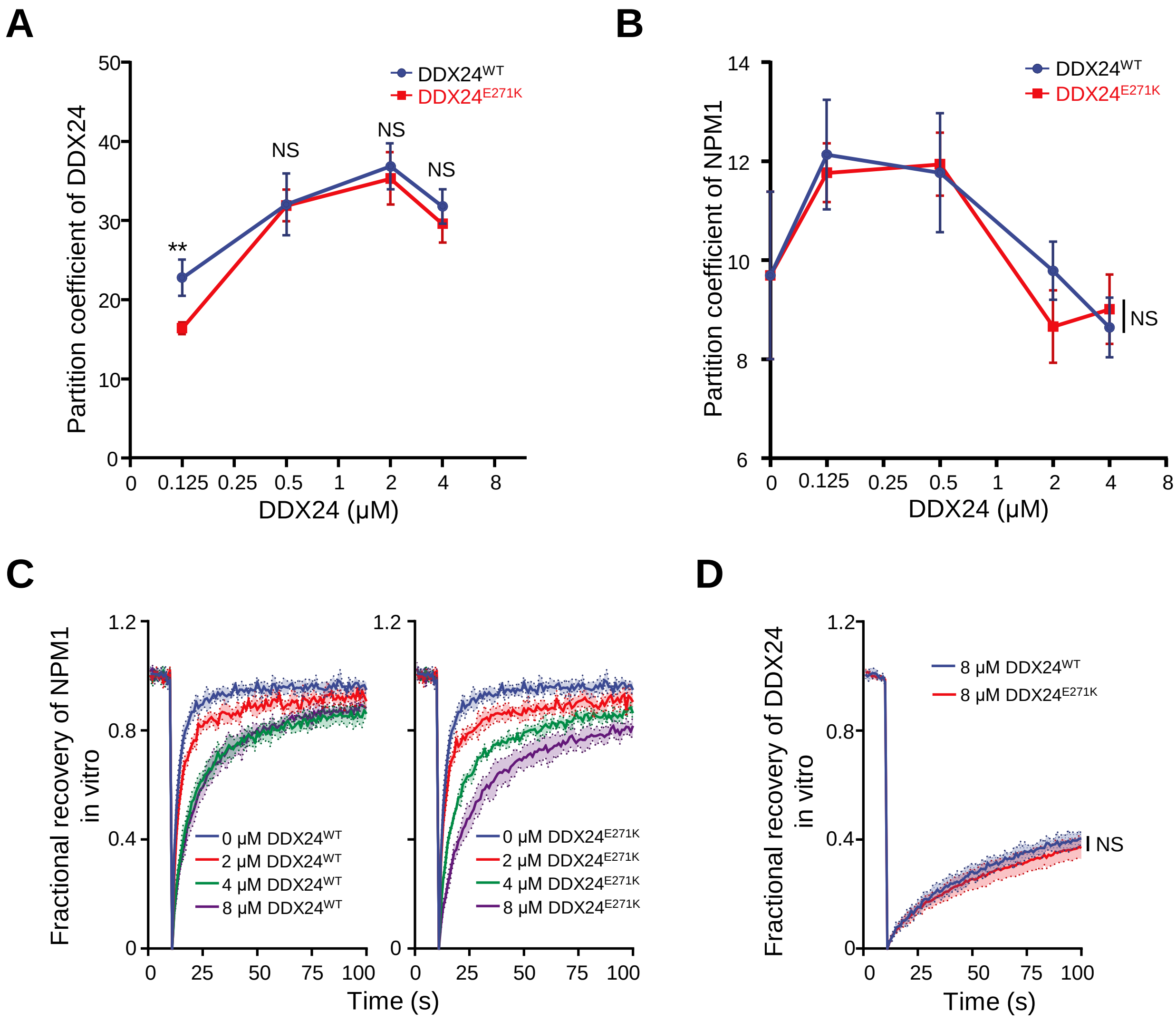

Supplemental Figure 8. LLPS parameters of DDX24 and NPM1 throughout the titration assays.

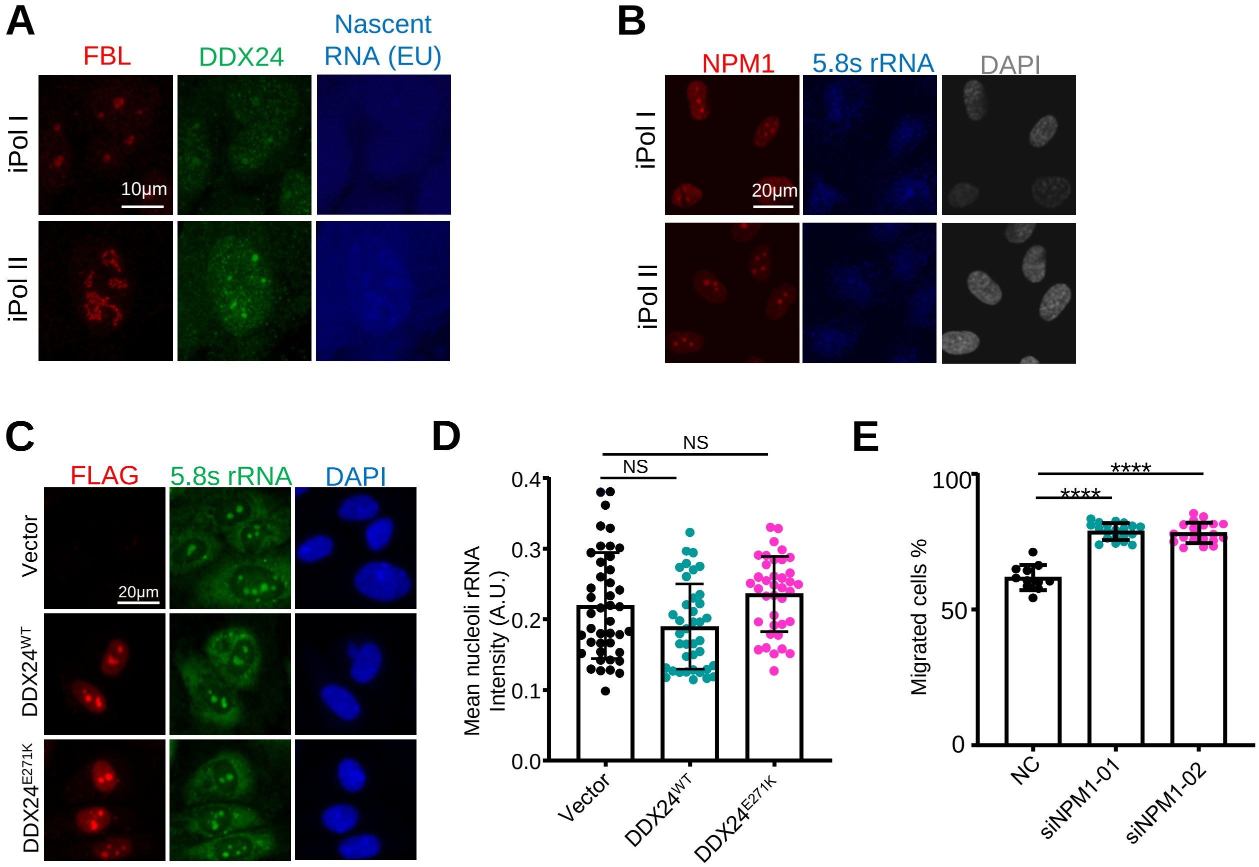

Supplemental Figure 9. Colocalization and functional assays in HUVECs.

Supplemental Table 1

SPR parameters calculated from SPR signal in **Fig. 3D-E** and **Fig.S5C-D**

SPR parameters	Association rates k_a (1/ M · s)	Dissociation rates k_d (1/s)	Binding constant K_D (M)
$DDX24^{WT}$ to NPM1	5.287×10^5	0.001261	2.386×10^{-9}
NPM1 to $DDX24^{WT}$	5.798×10^5	6.449×10^{-4}	1.112×10^{-9}
$DDX24^{E271K}$ to NPM1	5.749×10^5	0.003161	5.498×10^{-9}
NPM1 to $DDX24^{WT}$	5.635×10^5	0.001647	2.922×10^{-9}
